# North-south dipole in winter hydroclimate in the western United States during the last deglaciation

**DOI:** 10.1038/s41598-019-41197-y

**Published:** 2019-03-18

**Authors:** Adam M. Hudson, Benjamin J. Hatchett, Jay Quade, Douglas P. Boyle, Scott D. Bassett, Guleed Ali, Marie G. De los Santos

**Affiliations:** 1 U.S. Geological Survey, Denver, Colorado USA; 2 Division of Atmospheric Sciences, Desert Research Institute, Reno, Nevada USA; 3 Western Regional Climate Center, Reno, Nevada USA; 4 Department of Geosciences, University of Arizona, Tucson, Arizona USA; 5 Department of Geography, University of Nevada-Reno, Reno, Nevada USA; 6 Climate Change Institute, University of Maine, Orono, Maine USA; 7 AECOM Technical Services Inc., Minneapolis, Minnesota USA

## Abstract

During the termination of the last glacial period the western U.S. experienced exceptionally wet conditions, driven by changes in location and strength of the mid-latitude winter storm track. The distribution of modern winter precipitation is frequently characterized by a north-south wet/dry dipole pattern, controlled by interaction of the storm track with ocean-atmosphere conditions over the Pacific and Atlantic Oceans. Here we show that a dipole pattern of similar geographic extent persisted and switched sign during millennial-scale abrupt climate changes of the last deglaciation, based on a new lake level reconstruction for pluvial Lake Chewaucan (northwestern U.S.), and a compilation of regional paleoclimate records. This suggests the dipole pattern is robust, and one mode may be favored for centuries, thereby creating persistent contrasting wet/dry conditions across the western U.S. The TraCE-21k climate model simulation shows an equatorward enhancement of winter storm track activity in the northeastern Pacific, favoring wet conditions in southwestern U.S. during the second half of Heinrich Stadial 1 (16.1–14.6 ka) and consistent with paleoclimate evidence. During the Bølling/Allerød (14.6–12.8 ka), the northeastern Pacific storm track contracted poleward, consistent with wetter conditions concentrated poleward toward the northwest U.S.

## Introduction

### Deglacial hydroclimate in the western United States

**Figure 1:**
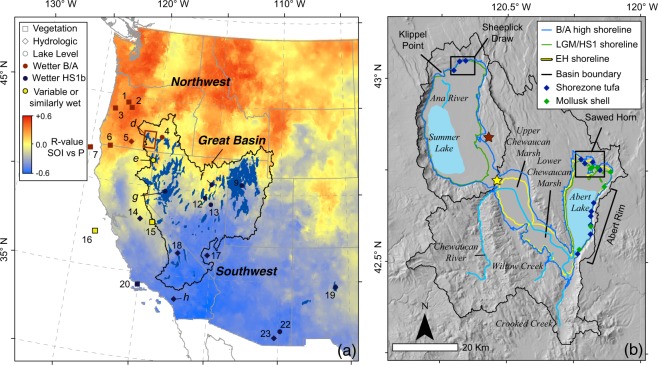
Western North America Study Area. (**a**) Modern winter hydroclimate and Deglacial paleoclimate records in western North America. Red-blue color ramp in the dipole map shows correlation coefficient (r) between modern (1926–2007) cool season (Oct-Mar) precipitation (P) and Jun-Nov Southern Oscillation Index (SOI); data from ref.^34^. Compilation of hydrologically sensitive paleoclimate records covering the Deglacial interval are plotted by climate indicator type and relative wetness during the HS1b or B/A periods (see legend). Full details and references for each numbered record are shown in the Methods and Table S1. Representative records lettered (**d**–**h)** are plotted in Fig. 2: (d) – Lake Chewaucan (this study, red box shows the map area of (**b))**, (e) – Lake Surprise^44^, (f) – Lake Franklin^52^, (g) – Lake Lahontan^53^, **(h)** – Lake Elsinore^4^. **(b)** Chewaucan drainage system. Drainage basin boundaries shown in black, approximate modern lake extents in light blue, B/A high shoreline extent shown in dark blue, LGM extent shown in green, early Holocene extent shown in yellow. ^14^C sample locations are plotted by sample type. Named areas where samples were collected are labeled (e.g. the Abert Rim). Overflow point (1338 m asl) connecting the Summer Lake sub-basin and Abert Lake sub-basin shown by yellow star. Location of shell ^14^C age sample from ref.^73^ that was omitted from our record is shown by red star, clearly well outside the high shoreline elevation (see Supplemental Information for details). Base hillshade was created using ArcGIS 10.4 using the 10 m-resolution digital elevation model from the USGS National Elevation Dataset^90^ (available from https://nationalmap.gov).

The drylands of the western United States experienced exceptionally wet climate conditions during the termination of the last glacial period (~18–11 ka; Deglacial, hereafter), evidenced by abrupt and dramatic changes in vegetation^1^, highstands of closed-basin lakes^2,3^, and increased discharge to lakes^4^ and desert wetlands^5^ (Fig. 1). This Deglacial interval was marked by several abrupt, millennial-scale climate shifts following the global Last Glacial Maximum (LGM, centered at 21 ka)^6^ that resulted in dramatic hydroclimate changes in tropical and mid-latitude regions worldwide^7^. The spatial pattern of hydrologic changes during these abrupt shifts can be reconstructed from networks of hydrologically sensitive paleoclimate records in the western U.S.^1,2,8,9^, and may provide insight into the evolution of regional climate change during this time of enhanced climate variability.

The first Deglacial climate shift coincided with cold Northern Hemisphere (NH) winter conditions during Heinrich Stadial 1 (HS1; 18.0–14.6 ka)^7^. This interval is often subdivided^10^ into an earlier (18.0–16.1 ka) and later period (16.1–14.6 ka; called HS1a, and HS1b, respectively hereafter). HS1b has been called the “Big Wet”^10^, because the highest closed-basin lake levels were achieved for most of the region^2,11^ during this interval, and many other paleohydrologic records indicate wet conditions^4,5,12^ (Fig. 1a). HS1a has also been called the “Big Dry”^10^. However, some paleoclimate records indicate wet conditions during both HS1a and HS1b^11^, defying a simple wet/dry pattern within the HS1 interval. Here we subdivide HS1 to be consistent with prior work, but do not retain these informal terms.

HS1 was followed by an abrupt change to warmer NH conditions during the Bølling/Allerød (B/A; 14.6–12.8 ka)^13^, resulting in drier or more variable conditions in most of the western U.S.^14^. The B/A interval was terminated by a return to cold NH winter conditions that occurred during the Younger Dryas (YD; 12.8–11.7 ka)^13^, during which desert wetlands expanded^5,15^, but other records indicate wet, similar or dry conditions relative to the B/A^4,16^. Many paleoclimate records in the western U.S. display paleohydrological shifts coinciding with this millennial-scale pattern. Yet, the spatial pattern of shifts towards wet or dry conditions during each climate shift is not uniform within the western U.S.^1,11,17^, and further work is needed to understand the climate forcings responsible for Deglacial hydroclimate change. In particular, paleoclimate records in the northwest U.S. indicate dry conditions during HS1 relative to those of the central and southern desert regions^1^. As a result, the source and seasonality of precipitation creating wet conditions in the western U.S. from the LGM to the B/A has been debated^1,10,18^.

Here we present a shoreline-based lake level record for pluvial Lake Chewaucan, a small closed-basin lake located in the northwest U.S. (42.5°N; Fig. 1a,b), that provides new constraint on regional moisture change during the Deglacial. This record displays opposing hydroclimate conditions to pluvial lake and other climate proxy records to its south during the HS1b and B/A intervals. To better understand the spatial pattern of regional moisture change during the Deglacial, we supplement our record with a compilation of regional paleoclimate proxy records sensitive to moisture change for the HS1b and B/A periods (Fig. 1a; Table S1). We pair this compilation with analysis of the TraCE-21k global climate model simulation^19,20^, focused on North Pacific winter storm track activity and zonally-averaged vertical circulations for time slices characteristic of these intervals, in order to better understand the atmospheric and oceanic controls on winter hydroclimate patterns. These metrics provide perspective on how past reorganizations of the global hydroclimate system were manifested on the landscape and controlled moisture availability in the western U.S. during the last deglaciation.

### Modern hydroclimate in the western U.S

Modern hydroclimate in the western U.S. is complex, and varies regionally in annual amount and seasonality of precipitation^21^. This complexity likely affected past hydroclimate, so that modern patterns may provide insight into what drove past changes. Annual precipitation is greatest overall in the northwest U.S., and occurs mainly during the cool season (October-March)^22^. In the southwest U.S., annual precipitation decreases, but an increasing summer season contribution from the North American Monsoon occurs as one moves southeastward into Arizona and New Mexico^21^. Effective moisture amount (the ratio of precipitation to evaporation) is mainly controlled by cool season precipitation, implying water limited conditions. The main source of runoff, soil moisture, and groundwater recharge is melting of accumulated winter snowfall^23^. Since most paleoclimate archives considered here record changes in annual-average effective moisture, we focus on modern winter climate conditions to help interpret past hydroclimate changes.

Winter hydroclimate in the western U.S. is strongly controlled by the mid-latitude westerly storm track that transports moisture from the Pacific Ocean via extratropical cyclones. Along the western margin of North America, precipitation associated with atmospheric rivers—narrow corridors of strong horizontal moisture transport located in the warm sector of extratropical cyclones^24^—contribute up to 50% of total precipitation during the cool season^25^. The central and eastern Great Basin also receives ~30% of annual precipitation from extratropical cyclones that are not associated with atmospheric river conditions during winter, as well as cutoff and closed low pressure systems during spring^26^ (March-June).

At interannual to decadal timescales winter hydroclimate often displays a north-south dipole in precipitation and runoff, wherein wet (dry) winters in the southwestern U.S. (SW, hereafter, corresponding to the SW dipole region in Fig. 1a) are mirrored by dry (wet) conditions in the northwestern U.S. (NW, corresponding to the NW dipole region) in a given year (Fig. 1a)^27,28^. This pattern is not always expressed, with some years showing anomalously wet or dry conditions across the whole region, but it is the leading mode of variability in regional winter precipitation at interannual and decadal timescales^22^. Extratropical cyclones with and without associated atmospheric river conditions contribute significantly to this dipole pattern, which characterizes the distribution of both total precipitation^22^ and extreme events^29^. The dipole is partly driven by Pacific and Atlantic ocean-atmosphere conditions linked to lower frequency modes of climate variability such as the El Niño Southern Oscillation (ENSO)^22,30^, the Pacific Decadal Oscillation (PDO)^31,32^ and the Atlantic Multidecadal Oscillation (AMO)^33,34^. These oscillations produce global rearrangements of sea surface temperatures, atmospheric pressure patterns, and tropical convection, which perturb the northern Pacific jet stream and storm track and ultimately influence the distribution and magnitude of western U.S. cool season precipitation. For example, during the warm (cool) phase of El Niño, as well as warm (cold) PDO and cold (warm) AMO conditions, moisture transport into the western U.S. is enhanced along an equatorward (poleward) track into the SW (NW)^32–34^. The modulation of the dipole by the PDO, which rather than a single dynamical mode is the collective oceanic response to coupled ocean-atmosphere interactions, appears to be related to changes in oceanic baroclinicity that shift storm tracks poleward (equatorward) during the negative (positive) phase^31^ and constructively enhances the ENSO teleconnections^34^.

These lower frequency teleconnection patterns interact with Arctic sea ice conditions and higher frequency atmospheric oscillations to further influence precipitation distributions by altering the location of landfalling storms and triggering additional changes in convection and atmospheric Rossby waves^35^. Such high frequency oscillations include the Pacific North America Pattern^36^, the Arctic Oscillation^37^, the Madden-Julien Oscillation^36^, and internal atmospheric variability^38^. The transition zone between the modern SW and NW dipole regions displays appreciable interannual variability, but despite the multiple factors influencing precipitation distributions, this area has remained relatively stationary at ~40°N across the western U.S. for the past 500 years^39^.

### The Lake Chewaucan shoreline record

**Figure 2:**
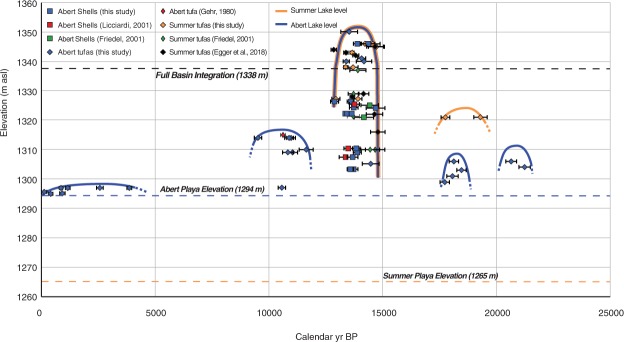
Lake Chewaucan lake level hydrograph, 0–25,000 cal yr BP. Abert Lake and Summer Lake playa elevations and threshold elevation connecting the Abert and Summer Lake sub-basins shown by dashed lines. Abert Lake (blue) and Summer Lake (gold) lake levels constrained by ^14^C ages shown by thick lines. Samples are denoted by differing symbols, and show the calibrated median ^14^C age and 2-sigma uncertainties. Shell ages come from this study (blue squares), ref.^43^ (red squares), and ref.^41^ (green squares). Tufa ages come from this study (blue and gold diamonds), ref.^41^ (green diamonds), ref.^44^ (black diamonds), and ref.^42^ (red diamond).

Pluvial Lake Chewaucan developed in a closed lake basin in the desert region of the NW, at the northwestern edge of the Great Basin (Fig. 1a). Modern annual precipitation in the basin peaks during the winter months, varying with elevation from 240 to 900 mm/yr^40^. Today, the closed drainage system terminates into two separate sub-basins, Summer Lake and Abert Lake, that were integrated at high lake level (>1338 m asl; Fig. 1b) to form Lake Chewaucan. Our lake level reconstruction for this system combines new shell and tufa ^14^C ages with those from previous work^41–45^ to date shoreline and nearshore lake deposits. Dated samples closely constrain lake surface elevation at the time of deposition (see Methods and Supplemental Information). The record spans intermittently from 21.3 ka to present. We interpret millennial-scale intervals with no ages to represent periods of lake regression below the elevation of our samples and/or subaerial exposure and erosion of deposits of that age (Fig. 2).

Three ages indicate Abert Lake level was moderately high ~21.0 ka, the lake receded from ~20.5–18.5 ka, then rose to nearly 1300 m asl from 18.4–17.8 ka. Two ages from the Summer Lake basin indicate a higher lake level of ~1324 m asl was achieved in this basin during the same ~21.0–18.0 ka interval. During the interval 17.8–14.6 ka, no ages were obtained on shoreline materials from either Summer or Abert Lake, suggesting a lake regression in both basins. This was followed by a rapid transgression to the high shoreline elevation (1350 m asl) at ~14.5 ka, as recorded by six overlapping tufa ages in both basins. Tufas of 14.5–13.4 ka age vary in elevation, indicating lake level may have fluctuated by ~30 m (Fig. S2), or that tufas formed at a range of elevations in the deep lake. However, in both sub-basins the deposits indicate lake level was mostly higher than the spilling threshold (1338 m asl), creating an integrated lake (Figs 1b and 2). All shell ages also fall within this interval. Shells collected near the highest shoreline elevations (1345–1350 m asl) agree well with the high shoreline tufa ages (~14.5 ka). All others have a narrow range of ages (~13.7 ka), consistent with the interpretation that the deposits represent a shell bed covering the lake bottom over a range of elevations, recording deep lake conditions in the Abert Lake sub-basin (see Methods and Supplemental Information).

Two ages on tufa, and one on a shell, indicate both lakes retreated below the spilling threshold by 12.9 ka, after which the lack of ages from 12.9–11.5 ka suggests the lakes receded below our sampled elevations. A lake transgression spanning 11.5–9.5 ka, reaching 1314 m asl, is recorded by four tufa ages on an extensive boulder shoreline exposed on the east side of the Abert sub-basin. No ages were obtained between 9.5 ka and 3.9 ka, indicating that Abert Lake receded to low lake level during the middle Holocene. In the Summer Lake sub-basin, the 7.6 ka Mazama tephra is found interbedded with dune deposits derived from deflation of the playa during the mid-Holocene^46^, in agreement with low Abert lake level. After this dry period, tufa ages from the playa along the Abert Rim indicate Abert lake level was similar to that of historical variability (<1300 m asl)^47^ intermittently from 3.9 ka to present (Fig. 2).

### Dipole-like hydroclimate change during the HS1-B/A transition

**Figure 3:**
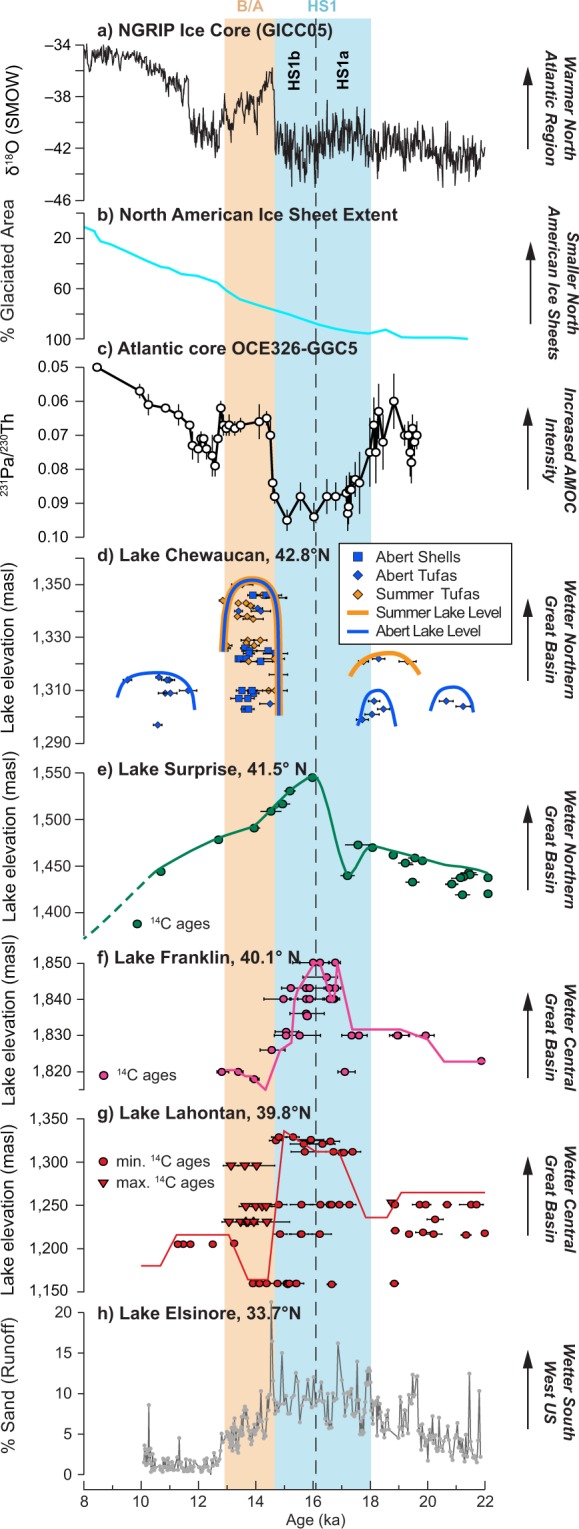
Representative Deglacial paleohydrologic records from western North America. (**a**) North Greenland Ice Core Project (NGRIP) oxygen isotope record^48^. (**b**) Areal extent of North American ice sheets^70^. (**c**) North Atlantic core OCE326-GGC5 Pa/Th (AMOC intensity)^65^. (**d**) Lake Chewaucan lake level and ^14^C ages, Abert sub-basin (blue) and Summer sub-basin (orange) shown separately. (**e**) Lake Surprise lake level based on ^14^C dating (green line; dashed where uncertain)^44^. (**f**) Lake Franklin lake level (pink, ^14^C ages in pink circles)^52^. (**g**) Lake Lahontan lake level (red, minimum limits on lake level red circles, maximum limits on lake level triangles)^53^. (**h**) Lake Elsinore percent sand-sized sediment (gray; proxy for watershed runoff)^4^. HS1 (18.0–14.6 ka) interval shown by blue bar, divided into HS1a (18.0–16.1 ka) and HS1b (16.1–14.6 ka) by dashed line. B/A interval (14.6–12.8 ka) shown by orange bar.

A striking aspect of our shoreline reconstruction is that the timing of deep lake conditions for Lake Chewaucan are out of phase with most closed lake systems in the western U.S. (Figs 1a and 3). No ^14^C ages fall within the HS1 interval (Fig. 3a)^48^, including the HS1b period when many other Great Basin closed lakes were deepest (Fig. 3e–g), and both wetlands^5^ and lake sediment (Fig. 3h)^4,49^ records indicate exceptionally wet conditions in the SW region. Instead, the deepest lake conditions at Lake Chewaucan developed abruptly at the beginning of the B/A and persisted throughout the interval (Fig. 3d). This pattern is more similar to records found in the NW, where a shift from dry climate to wetter conditions occurs in many pollen-based vegetation records north of Chewaucan at ~14.5 ka^50^ (Fig. 1a; Table S1). These climate conditions are also expressed in the diatom stratigraphy of Klamath Lake, just ~100 km to the west^51^. Overall, the records in our compilation (Fig. 1a) define a map pattern of drier (wetter) conditions during HS1b (B/A) in the NW, in contrast to wetter HS1b (drier B/A) conditions in most records south of 40°N. This pattern is similar in spatial extent to the winter precipitation dipole that characterizes interannual climate variability in the modern instrumental record (Fig. 1a)^22,34^. While the overall dipole pattern during this HS1-B/A shift is well supported by paleoclimate evidence, the transition zone exhibits a minor shift in latitude compared to the modern pattern. The deepest conditions observed for pluvial Lake Surprise coincide with HS1b, resembling the lakes in the SW region more than nearby Chewaucan (Fig. 3e)^44^. At 41.5°N, Lake Surprise, along with Lake Chewaucan, is located in the modern transition zone of the dipole pattern (Fig. 1a). Unlike Lake Chewaucan to the north (Fig. 3d, this study) and lakes to its south (Fig. 3f, g)^52,53^, Lake Surprise remained >100 m deeper than the modern level throughout HS1, the B/A and YD, consistent with a transitional position. This suggests the present transition zone was shifted to the south of Chewaucan, but remained over Lake Surprise during the Deglacial. Despite small shifts in transition latitude, paleoclimate evidence strongly supports a persistent dipolar precipitation pattern similar in spatial extent to that expressed in modern interannual precipitation variability^34^.

In addition to the HS1-B/A interval, a dipole pattern of similar extent is visible in compilations of LGM paleoclimate records^8,9,54^ (21 ka), indicating winter storms are displaced to similar northerly or southerly mean tracks under full glacial, Deglacial millennial-scale (this study), and modern conditions^39^. There is also some evidence a dipole-like pattern appears coincident with earlier stadial/interstadial climate shifts. Previous records have identified abrupt, millennial-scale moisture changes associated with Dansgaard-Oeschger (D-O) cycles during MIS 2–3, with moist (dry) conditions inferred in the NW during interstadials (stadials)^51,55^, balanced by moist conditions during D-O and Heinrich stadials recorded by wetlands and lake sediments in the SW^5,56^. However, other records have noted wet D/O interstadial conditions as far south as 34 °N closer to the west coast^57^, suggesting this regional pattern may differ from a strict north-south dipole during prior millennial-scale changes.

### Ice sheet and AMOC forcing of Deglacial storm track position

It has been widely suggested that the westerly storm track shifted equatorward in mean winter position and/or strengthened, bringing more frequent or wetter winter storms to the western U.S. during the LGM and Deglacial periods^8,9,58–60^. However, the relative importance of the forcing mechanism(s) causing these storm track shifts are less well constrained. Early hypotheses^61^ and climate model simulations^58^ focused on the atmospheric effect of the Laurentide Ice Sheet (LIS). Recent model/record comparisons have confirmed that persistent high atmospheric pressure over the LIS likely affected the storm track, favoring more southerly incidence of storms into the western U.S., and creating a dry-NW/wet-SW dipole pattern relative to modern climate during peak LGM conditions (21 ka)^8,9^. Likewise, rapid melting and partial collapse of the LIS and Cordilleran Ice Sheet during the B/A (Fig. 3b) reduced this ice sheet perturbation, creating a poleward shift in the storm track^16,18^ and moisture delivery^18^, contributing to the wet-NW/dry-SW mode recorded by Lake Chewaucan and other records after the HS1-B/A transition (Figs 1a and 3). The LIS also likely continued to perturb atmospheric circulation into the early Holocene (>8 ka), maintaining wetter than modern conditions in the western U.S. despite peak Holocene summer insolation^62^. Ice sheet topography clearly was an important driver of storm track changes across the Deglacial interval. However, ice sheet changes do not explain the correspondence between the dipole-like shift in moisture in the western U.S. and North Atlantic-driven millennial scale climate changes (Fig. 3), which are known to have caused similar meridional shifts in circulation patterns in both tropical^63,64^ and mid-latitude regions across the globe^7^.

Previous work^7^ has suggested that variation in the interhemispheric meridional temperature gradient (centered at the thermal equator, or region of maximum mean average temperature), driven largely by Northern Hemisphere warming and Arctic sea ice change, may also influence the westerly storm track, and may explain the correspondence between millennial-scale dipole switches and global circulation shifts. The wettest conditions in the SW region records largely coincided with HS1b, displaying a dry-NW/wet-SW dipole pattern (Figs 1a and 3). During this time, massive freshwater discharge into the North Atlantic culminated in a major reduction of the Atlantic Meridional Overturning Circulation (AMOC; Fig. 3c)^65^. This led to millennial-scale cooling of the Arctic (Fig. 3a)^48^ and southward shifts in precipitation-bringing circulation patterns in both hemispheres^7,63,64^. The shift to a wet-NW/dry-SW pattern at ~14.5 ka coincides with abrupt strengthening of AMOC, NH warming, and a northward shift in global precipitation patterns, consistent with a northward shift in the thermal equator^7^. This close correspondence suggests precipitation changes in the western U.S. form part of a larger global pattern in circulation changes that extend beyond ice sheet influence on the Pacific storm track.

To examine the global circulation dynamics that may have contributed to the observed HS1-B/A dipole shift, we compare storm track activity and zonally averaged vertical motions calculated from two 50-year time slices of daily output from the TraCE-21k transient climate model simulation (see Methods)^19^ for AMOC shutdown conditions during HS1 (17.00 ka) and the resumption during the B/A (14.36 ka). Importantly, the B/A timeslice used here (14.36 ka) simulates conditions before the major ice sheet saddle collapse^18^, prescribed at 13.87 ka in the simulation^19^, to investigate the effect of AMOC-driven change on storminess. It has been noted that TraCE-21k underestimates the increase in precipitation required to create the deep lake conditions of the Deglacial^11^. However, it does reproduce the general spatial pattern of moisture change through the Deglacial interval seen in paleoclimate records^16,18^. The coarse resolution of global atmospheric models has been shown to introduce biases into important finer scale processes such as atmospheric rivers^66^. This renders the 3.25° horizontal resolution TraCE-21k insufficient to robustly assess moisture delivery in the present study. Nonetheless, as the only publicly available model with daily temporal resolution for the HS1 and B/A time slices, TraCE-21k still offers the ability to explore planetary-scale dynamics that accompanied abrupt climate change during the deglaciation.

**Figure 4:**
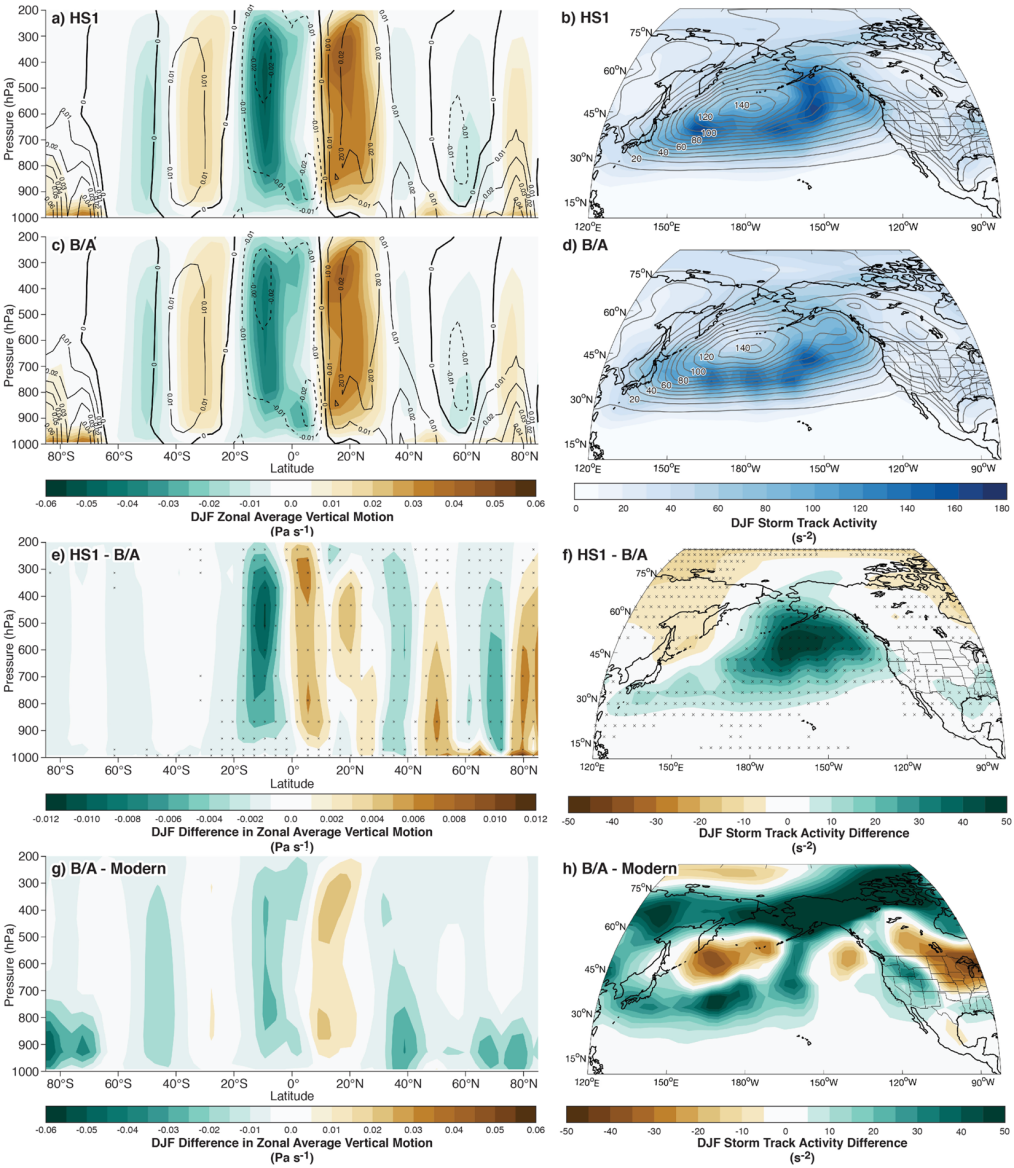
TraCE-21k climate model analyses for HS1 (17.00 ka) and B/A (14.36 ka) periods. December-February zonal averaged vertical motion (Pa s^−1^) for HS1 (**a**), B/A (**c**), and their difference (HS1 minus B/A) (**e**) shown by brown (positive, downward motion) to green (negative, upward motion) filled contours. December-February storm track activity (s^−2^) over the Pacific Ocean for HS1 (**b**), B/A (**d**) shown by blue filled contours. Storm track differences for (HS1-B/A) (**f**) shown by brown (negative, B/A greater than HS1) to green (positive, HS1 greater than B/A) filled contours. NCEP/NCAR^89^ 1981–2010 averages for December-February vertical motion and storm track activity are shown as labeled open contours in the same units for each plot (**a**–**d**) (negative values are dashed in (**a**,**c**)). Statistically significant differences between the gridpoints at the alpha = 0.01 level are noted by stippling. Difference in vertical motions (**g**) and storm track activity (**h**) between the B/A from TraCE-21k output and the modern reanalysis observations^89^ are displayed as (**g**,**h**) above for zonal averaged vertical motion and storm track activity, respectively.

Comparing global zonal-average atmospheric vertical motion (a measure of the location of major meridional circulation cells) in TraCE-21k shows a southward shift during HS1 relative to the B/A (Fig. 4a,c,e). This is consistent with a southward shift in the winter thermal equator under stadial conditions and a northward shift during the B/A interstadial^7^. Winter season (December-February) storm track activity (a measure of surface atmospheric pressure variance associated with the passage of extratropical cyclones) between HS1 and the B/A in TraCE-21k does not show a wholesale equatorward shift during HS1 (Fig. 4b,d,f). Instead, storm track activity is intensified and expanded equatorward during HS1 relative to the B/A across the north Pacific with the largest changes centered about the Aleutian Low region (Fig. 4f). This intensification and southward expansion of surface pressure variance in the Pacific basin is similar to the results of recent hosing experiments comparing LGM and HS1 conditions in the region^11^. In their simulation, AMOC shutdown conditions were characterized by a southeastward shifted and deepened Aleutian Low, and an intensified and equatorward shifted Pacific jet stream. These changes drove enhanced southwesterly wind anomalies and moisture transport from subtropical latitudes into the SW region. Moisture transport occurs primarily along the equatorward side of storm track activity maxima^9,67^, so the southward expansion and enhancement in storminess during HS1 (relative to the B/A time interval) appears consistent with an increased frequency of moisture-rich atmospheric river-type storms impacting the SW region^11^. Since the SW is significantly drier overall, the precipitation contribution of these intense events makes up a much greater proportion of total seasonal precipitation^25^ and drives the interannual variability in this region^68^. While simulated changes in northern Pacific storm track activity do not demonstrate a uniform equatorward shift, our results suggest an increased frequency of intense storms in the SW region as indicated by the southward-expanded storm track. This is broadly consistent with the southward shift in the intertropical convergence zone identified in hosing experiments^11,60^ and is captured by TraCE-21k (Fig. 4a,c,e). The increase in southwesterly moisture transport favored by the deepened Aleutian Low^11^ (Fig. 4b) and stronger subtropical jet stream^59^ during HS1 may also have facilitated more frequent inland penetration of atmospheric rivers into the interior SW region through the Mojave Desert^69^.

Relative to modern conditions, enhanced storm track activity during the B/A interval is also shifted equatorward over the North Pacific and southeastward over western North America consistent with a southward shift in zonal-average vertical motions during this time (Fig. 4g,h). This indicates that although the B/A average storm track was weaker and contracted poleward relative to HS1, it was still strengthened and further equatorward than that of the Holocene interglacial conditions. This likely reflects the effect of the large, but melting (Fig. 3b)^70^ North American ice sheets on the storm track^18^, which favored more frequent and/or intense storms in the NW and promoted the exceptionally wet conditions (relative to modern) recorded by the highstand of Lake Chewaucan during the B/A.

## Conclusions

The record of lake level fluctuations for Lake Chewaucan, located in the northern Great Basin, indicates peak wet conditions began abruptly at the start of the B/A period (14.6–12.8 ka) and persisted throughout the interval. This follows the HS1b period (16.1–14.6 ka) when other major closed basin lake systems indicate peak wetness, and occurs while lakes to the south dried. Based on a compilation of paleoclimate records for the region, this forms a pattern of north-south wet/dry conditions of similar spatial extent to the dipole observed in modern interannual winter precipitation. For the western U.S., the modern regional dipole pattern in winter precipitation is most clearly related to ENSO variability in the tropical Pacific^22^ while being modulated by other modes of low and high frequency coupled atmosphere-ocean variability. However, our new lake level reconstruction for Lake Chewaucan, in conjunction with other paleoclimate records, clearly demonstrates that on millennial timescales, variations in global circulation associated with AMOC strength can favor the persistence of one average dipole state for hundreds to thousands of years. During the last deglaciation, these paleoclimate records clearly record exceptionally wet conditions in the western U.S., coupled with abrupt shifts in the regions of wet and dry conditions corresponding with North Atlantic-forced millennial-scale climate changes. Global climate model simulations of abrupt climate events during the Deglacial are also consistent in suggesting a combination of atmospheric forcing by large North American ice sheets and changes in zonal-mean meridional circulations that altered North Pacific storm track and moisture delivery and created the hydroclimatic dipole signature. We recommend that continuing research on the millennial-scale Deglacial dipole focus on the development of lake level (or other proxy) records along the transition region (40 °N) as well as model studies performed at horizontal and vertical resolutions sufficient to capture key processes driving the magnitude and distribution of precipitation.

## Methods

### Field and laboratory sampling

Sample localities and stratigraphic sections in the study area were sampled during 2013–2015. Tufas were sampled *in situ* from bedrock outcrops or “in place” shoreline clasts using a hammer and chisel (Fig. S1). Mollusk shells were sampled *in situ* from natural exposures or hand-dug trenches (Fig. S3). The stratigraphy of the sediments was then described in detail (Fig. S4). Each sample location was recorded using a high-precision differential GPS unit. In the laboratory, we sampled tufas and mollusk shells following established methods^45^, targeting the densest tufa material and abrading the outer surface of samples that were exposed to subaerial weathering in order to minimize secondary contamination since tufa deposition. Single intact shells were used for ^14^C dating in order to avoid combining material of different ages.

### ^14^C dating

Each tufa or shell sample was reacted with 100% H_3_PO_4_ and vacuum-extracted and purified^69^. AMS and δ^13^C measurements were performed by the Arizona AMS Laboratory. Raw ^14^C ages are reported with 1σ uncertainty and were calibrated using the OxCal 4.2 software^71^ with the IntCal13 calibration curve^72^. All ages aggregated from previous publications^41–45^ have been recalibrated using IntCal13 for consistency. All calibrated ^14^C ages are reported in calendar years (cal yr BP) or calendar kiloyears before present (ka) as the median age of the calendar range plus or minus 2σ uncertainty.

### Constructing the Chewaucan lake level record

The lake level record is based on 73 ^14^C ages of shorezone tufa samples collected on bedrock outcrops and boulder paleo-shoreline deposits, and shells of mollusks (*Vorticifex effusa, Pyrgylopsis* sp, and *Valvata humeralis*) that lived in shallow water habitats. We have omitted one previously reported shell age^73^, due to lack of information on the sample material and inconsistency between its reported location and elevation (see Supplemental Information).

For the lake level reconstruction, the depositional context (below water) of all sampled materials indicates sample elevation is a minimum estimate for lake surface elevation. Tufa forms at multiple paleo-shoreline levels on shoreline facies gravels in both sub-basins in areas with modern or paleo-spring discharge along faults. Their appearance on coarse shoreline gravels on high paleo-shorelines and on the modern shoreline of Lake Abert indicates they were largely deposited at only a few meters depth. *V. effusa* are freshwater pulmonate mollusks that feed on algae on rocky substrates like the Lake Chewaucan shorelines^74^, indicating they formed shells in shallow water. The other taxa live on aquatic vegetation, also in the near-shore environment^74^. All fossil shells were collected from the Abert Lake sub-basin, and of these, the majority were deposited in a 5–10 cm thick coquina in near-shore sandy deposits lower in elevation and distal to the shoreline gravels (Fig. S3). The coquina deposit is a conspicuous marker bed within the Abert Lake basin that comprises shells that were reworked from their natural habitat on the shorelines. Shells derived from this bed have also been reworked into gravels of lower shorelines, which are deposited unconformably on the coquina (Fig. S4, section CHL13-19). In both circumstances, significant reworking of shells is indicated. Based on these depositional characteristics, we regard the shorezone tufas to represent the best estimate of past lake level, while the shells provide minimum lake level estimates. Due to semi-continuous sedimentation into the basins since Deglacial time, all lake depths measured versus the modern playas are also regarded as minima. Normal faulting within the basin cuts shorelines and lake deposits of late Pleistocene age, creating scarps of 4–5 m height^75^, suggesting some variation in sample elevation may be imparted by tectonic uplift or drop since deposition, which may explain some variation in sample elevation within deep lake phases.

The reservoir effect within the lakes has been assessed by dating modern shorezone tufas and lake DIC, yielding bomb carbon-equilibrated ages (Table S2). In addition, previous work^70^ indicates the carbon isotope composition of tufa is invariant regardless of past lake volume and consistent with inorganic precipitation from lake water DIC in equilibrium with pre-industrial atmospheric CO_2_. This finding indicates the lake epilimnion was well mixed with the atmosphere at all times in the past so that tufas and shells of shallow-water mollusk taxa should yield accurate ^14^C ages. Shells are composed of 100% aragonite (based on XRD analyses of select samples)^70^ indicating post-depositional weathering has not affected them. Ages from tufa and shell are in good agreement during the deepest lake interval, providing additional confidence in the age dataset. Based on this evidence, we applied no reservoir correction.

### Compilation of paleohydrologic records

The compilation of 23 total paleohydrologic records (Table S1; Fig. 1a) were included for comparison to Lake Chewaucan and the TraCE-21k analysis. These include pollen-based vegetation records^1,50,76,77^, shoreline-based lake level records^44,52,53,78–86^, lacustrine fauna (diatoms, ostracoda) moisture records^49,51,82^, other lake sediment-based hydrologic proxies^4,87^, and desert wetlands discharge records^5,15^. This compilation was chosen based on several criteria; the first being that they were sensitive primarily to effective moisture amount. Records that are less sensitive to effective moisture amount, such as glacier records (which respond strongly to temperature), and speleothem stable isotope records (which are also sensitive to temperature, moisture source, and seasonality) were omitted. This is with the exception of McLean’s and Moaning Cave from the central Sierra Nevada, where trace element data were coupled with oxygen isotope data to provide a more robust hydrological signal^12^.

Records were also chosen based on their age model uncertainty, and the timespan covered during the Deglacial interval. Since the focus of our analysis was on the HS1b and B/A periods, continuous records (i.e. core reconstructions) needed to cover the entirety of both intervals. Continuous records also needed sufficiently low age model uncertainty (generally ~400 calendar years, Table S1) to clearly differentiate millennial-scale changes. For example, many pollen-based vegetation records produced before electronic datasets were widely available (so only the publication graphic of the record is available) and before routine calibration of ^14^C age timescales, were omitted due to inability to translate those records objectively to a timescale comparable to more recent records. As the offset between calendar and ^14^C years varies considerably between >1000 and >2000 years during the Deglacial interval^72^, these records have too much uncertainty to reliably define millennial scale changes. Age model uncertainty was estimated as the average 2 sigma uncertainty for all ages (^14^C, U-Th series, or curve-matching) falling within the B/A and HS1 intervals (18.0–12.8 ka in total). Definition of relative moisture conditions during each millennial scale interval was based on the interpretations of proxy evidence by the original authors.

Inherently discontinuous records (i.e. shoreline records, wetlands sediment records) were chosen based on the occurrence of evidence of moist conditions at some part of the Deglacial interval, and required at least three ages within the interval of interest in order to be included (Table S1). Age model uncertainty was estimated identically to that for continuous records. Relative wetness during each millennial-scale interval with ages was defined by the interpretations of the original authors. For example, the lake level reconstruction for Jakes Lake only contains evidence of significant deep lake conditions during the HS1 interval, with the authors interpreting lack of lake deposits of other ages to indicate regression of the lake to lower levels.

### TraCE-21k analysis methods

We used daily output from the 3.25° horizontal resolution and 16 isobaric level TraCE-21k global climate model simulation^19,20^ to calculate storm track activity and vertical motions. Fifty-year time slices were used at 17.00 ka for HS1 and 14.36 ka for the B/A. Storm track activity (Fig. 4b,d,f) was calculated as the 24-hour, first-differenced sea level pressure variance and averaged for meteorological winter (December to February)^67,88^. Model-derived vertical motions at daily time steps during the 50-year time periods were used to estimate the mean winter position of the zonal-averaged meridional circulation and serve as a proxy for the position of the intertropical convergence zone (Fig. 4a,c,e). Statistically significant differences between the two time periods was estimated by requiring each grid point to satisfy both a two-sided Student’s t-test for differences in mean values and a Kolmogorov-Smirnoff test for differences in distributions. An alpha level of 0.01 was used for both calculations. The modern (1981–2010) storm track activity and circulation, displayed for reference (Fig. 4b,d), was calculated using 24-hour, first-differenced sea level pressure variance from the 2.5° horizontal resolution and 26 isobaric level NCEP-NCAR reanalysis^89^. Simulated vertical motions were also obtained from the NCEP-NCAR reanalysis. NCEP-NCAR output was re-gridded to the resolution of TraCE-21k for calculation of differences between TraCE-21k vertical motions (Fig. 4g) and storm track activity (Fig. 4h) during the B/A and the modern (1981–2010) period.

## Supplementary information

Supplemental Information

## References

[1] Lyle M (2012). Out of the Tropics: The Pacific, Great Basin Lakes, and Late Pleistocene Water Cycle in the Western United States. Science.

[2] Munroe JS, Laabs BJC (2013). Temporal correspondence between pluvial lake highstands in the southwestern US and Heinrich Event 1. J. Quat. Sci..

[3] Reheis MC, Adams KD, Oviatt CG, Bacon SN (2014). Pluvial lakes in the Great Basin of the western United States—a view from the outcrop. Quat. Sci. Rev..

[4] Kirby ME (2018). A late Wisconsin (32-10 k cal a BP) history of pluvials, droughts and vegetation in the Pacific south-west United States (Lake Elsinore, CA): California’s Glacial History of Pluvials, Droughts and Vegetation. J. Quat. Sci..

[5] Springer KB, Manker CR, Pigati JS (2015). Dynamic response of desert wetlands to abrupt climate change. Proc. Natl. Acad. Sci..

[6] Clark PU (2009). The Last Glacial Maximum. Science.

[7] Broecker WS, Putnam AE (2013). Hydrologic impacts of past shifts of Earth’s thermal equator offer insight into those to be produced by fossil fuel CO_2_. Proc. Natl. Acad. Sci..

[8] Oster JL, Ibarra DE, Winnick MJ, Maher K (2015). Steering of westerly storms over western North America at the Last Glacial Maximum. Nat. Geosci..

[9] Lora, J. M., Mitchell, J. L., Risi, C. & Tripati, A. E. North Pacific atmospheric rivers and their influence on western North America at the Last Glacial Maximum. *Geophys. Res. Lett*. **44** (2017).

[10] Broecker W, Putnam AE (2012). How did the hydrologic cycle respond to the two-phase mystery interval?. Quat. Sci. Rev..

[11] McGee D, Moreno-Chamarro E, Marshall J, Galbraith ED, Western US (2018). lake expansions during Heinrich stadials linked to Pacific Hadley circulation. Sci. Adv..

[12] Oster JL (2015). Stalagmite records of hydroclimate in central California during termination 1. Quat. Sci. Rev..

[13] Clark PU (2012). Global climate evolution during the last deglaciation. Proc. Natl. Acad. Sci..

[14] Shuman BN, Serravezza M (2017). Patterns of hydroclimatic change in the Rocky Mountains and surrounding regions since the last glacial maximum. Quat. Sci. Rev..

[15] Pigati JS, Bright JE, Shanahan TM, Mahan SA (2009). Late Pleistocene paleohydrology near the boundary of the Sonoran and Chihuahuan Deserts, southeastern Arizona, USA. Quat. Sci. Rev..

[16] Wong CI (2016). Evolution of moisture transport to the western U.S. during the last deglaciation: past hydroclimate dynamics to the western U.S. Geophys. Res. Lett..

[17] Kirby ME, Feakins SJ, Bonuso N, Fantozzi JM, Hiner CA (2013). Latest Pleistocene to Holocene hydroclimates from Lake Elsinore, California. Quat. Sci. Rev..

[18] Lora JM, Mitchell JL, Tripati AE (2016). Abrupt reorganization of North Pacific and western North American climate during the last deglaciation. Geophys. Res. Lett..

[19] He, F. Simulating transient climate evolution of the last deglaciation with CCSM3. (University of Wisconsin-Madison, 2011).

[20] Liu Z (2012). Younger Dryas cooling and the Greenland climate response to CO_2_. Proc. Natl. Acad. Sci..

[21] Guirguis KJ, Avissar R (2008). A Precipitation Climatology and Dataset Intercomparison for the Western United States. J. Hydrometeorol..

[22] Dettinger MD, Cayan DR, Diaz HF, Meko DM (1998). North–South Precipitation Patterns in Western North America on Interannual-to-Decadal Timescales. J. Clim..

[23] Flint LE, Flint AL (2007). Regional analysis of ground-water recharge. In Ground-water recharge in the arid and semiarid southwestern United States.

[24] Ralph FM, Neiman PJ, Wick GA (2004). Satellite and CALJET aircraft observations of atmospheric rivers over the eastern North Pacific Ocean during the winter of 1997/98. Mon. Weather Rev..

[25] Rutz JJ, Steenburgh WJ, Ralph FM (2014). Climatological characteristics of atmospheric rivers and their inland penetration over the western United States. Mon. Weather Rev..

[26] Abatzoglou JT (2016). Contribution of Cutoff Lows to Precipitation across the United States. J. Appl. Meteorol. Climatol..

[27] Redmond KT, Koch RW (1991). Surface Climate and Streamflow Variability in the Western United States and Their Relationship to Large-Scale Circulation Indices. Water Resour. Res..

[28] Lins HF (1997). Regional streamflow regimes and hydroclimatology of the United States. Water Resour. Res..

[29] Jiang P, Yu Z, Gautam MR (2013). Pacific and Atlantic Ocean influence on the spatiotemporal variability of heavy precipitation in the western United States. Glob. Planet. Change.

[30] Kim, H.-M., Zhou, Y. & Alexander, M. A. Changes in atmospheric rivers and moisture transport over the Northeast Pacific and western North America in response to ENSO diversity. *Clim. Dyn*., 10.1007/s00382-017-3598-9 (2017).

[31] Sung M-K, An S-I, Kim B-M, Woo S-H (2014). A physical mechanism of the precipitation dipole in the western United States based on PDO-storm track relationship: Precipitation dipole in the western U.S. Geophys. Res. Lett..

[32] Gershunov A, Shulgina T, Ralph FM, Lavers DA, Rutz JJ (2017). Assessing the climate-scale variability of atmospheric rivers affecting western North America: Atmospheric River Climate-Scale Behavior. Geophys. Res. Lett..

[33] McCabe GJ, Palecki MA, Betancourt JL (2004). Pacific and Atlantic Ocean influences on multidecadal drought frequency in the United States. Proc. Natl. Acad. Sci..

[34] Wise, E. K. Spatiotemporal variability of the precipitation dipole transition zone in the western United States. *Geophys. Res. Lett*. **37** (2010).

[35] Cvijanovic, I. *et al*. Future loss of Arctic sea-ice cover could drive a substantial decrease in California’s rainfall. *Nat. Commun*. **8** (2017). 10.1038/s41467-017-01907-4 PMC5717256 29209024

[36] Guan B, Waliser DE (2015). Detection of atmospheric rivers: Evaluation and application of an algorithm for global studies: Detection of Atmospheric Rivers. J. Geophys. Res. Atmospheres.

[37] Guan B, Molotch NP, Waliser DE, Fetzer EJ, Neiman PJ (2013). The 2010/2011 snow season in California’s Sierra Nevada: Role of atmospheric rivers and modes of large-scale variability: Atmospheric Rivers and Modes of Large-Scale Variability. Water Resour. Res..

[38] Coats S (2016). Internal ocean-atmosphere variability drives megadroughts in Western North America: Internal Variability Drives Megadroughts. Geophys. Res. Lett..

[39] Wise EK (2016). Five centuries of U.S. West Coast drought: Occurrence, spatial distribution, and associated atmospheric circulation patterns: Five Centuries of West Coast Drought. Geophys. Res. Lett..

[40] Daly C (2008). Physiographically sensitive mapping of climatological temperature and precipitation across the conterminous United States. Int. J. Climatol..

[41] Friedel, D. Pleistocene Lake Chewaucan: two short pieces on hydrological connections and lake-level oscillations. *In Quaternary Studies near Summer Lake, Oregon* (2001).

[42] Gehr, K. D. Late Pleistocene and recent archaeology and geomorphology of the south shore of Harney Lake, Oregon. (Portland State University, 1980).

[43] Licciardi JM (2001). Chronology of latest Pleistocene lake-level fluctuations in the pluvial Lake Chewaucan basin, Oregon, USA. J. Quat. Sci..

[44] Egger, A. E. *et al*. Influence of pluvial lake cycles on earthquake recurrence in the northwestern Basin and Range, USA. In *Special Paper 536: From Saline to Freshwater: The Diversity of Western Lakes in Space and Time* **536** (Geological Society of America, 2018)

[45] Hudson AM (2017). Stable C, O and clumped isotope systematics and ^14^C geochronology of carbonates from the Quaternary Chewaucan closed-basin lake system, Great Basin, USA: Implications for paleoenvironmental reconstructions using carbonates. Geochim. Cosmochim. Acta.

[46] Kuehn SC, Negrini RM (2010). A 250 k.y. record of Cascade arc pyroclastic volcanism from late Pleistocene lacustrine sediments near Summer Lake, Oregon, USA. Geosphere.

[47] Phillips KN, Van Denburgh AS (1971). Hydrology and Geochemistry of Abert, Summer, and Goose Lakes, and Other Closed-Basin Lakes in South-Central Oregon. US Geol. Surv. Prof. Pap..

[48] Svensson A (2008). A 60 000 year Greenland stratigraphic ice core chronology. Clim. Past.

[49] Menking KM, Polyak VJ, Anderson RY, Asmerom Y (2018). Climate history of the southwestern United States based on Estancia Basin hydrologic variability from 69 to 10 ka. Quat. Sci. Rev..

[50] Briles CE, Whitlock C, Bartlein PJ (2005). Postglacial vegetation, fire, and climate history of the Siskiyou Mountains, Oregon, USA. Quat. Res..

[51] Bradbury JP, Colman SM, Dean WE (2004). Limnological and Climatic Environments at Upper Klamath Lake, Oregon during the past 45 000 years. J. Paleolimnol..

[52] Munroe JS, Laabs BJC (2013). Latest Pleistocene history of pluvial Lake Franklin, northeastern Nevada, USA. Geol. Soc. Am. Bull..

[53] Benson LV (2013). Insights from a synthesis of old and new climate-proxy data from the Pyramid and Winnemucca lake basins for the period 48 to 11.5 cal ka. Quat. Int..

[54] Morrill C, Lowry DP, Hoell A (2018). Thermodynamic and Dynamic Causes of Pluvial Conditions During the Last Glacial Maximum in Western North America: LGM Western U.S. Moisture Budget. Geophys. Res. Lett..

[55] Zic M, Negrini RM, Wigand PE (2002). Evidence of synchronous climate change across the Northern Hemisphere between the North Atlantic and the northwestern Great Basin, United States. Geology.

[56] Reheis MC (2015). Directly dated MIS 3 lake-level record from Lake Manix, Mojave Desert, California, USA. Quat. Res..

[57] Glover KC (2017). Evidence for orbital and North Atlantic climate forcing in alpine Southern California between 125 and 10 ka from multi-proxy analyses of Baldwin Lake. Quat. Sci. Rev..

[58] COHMAP Members (1988). Climatic Changes of the Last 18,000 Years: Observations and Model Simulations. Science.

[59] Hostetler S, Benson LV (1990). Paleoclimatic implications of the high stand of Lake Lahontan derived from models of evaporation and lake level. Clim. Dyn..

[60] Chiang JCH, Lee S-Y, Putnam AE, Wang X (2014). South Pacific Split Jet, ITCZ shifts, and atmospheric North–South linkages during abrupt climate changes of the last glacial period. Earth Planet. Sci. Lett..

[61] Antevs E (1945). Correlation of Wisconsin glacial maxima. Am. J. Sci..

[62] Steponaitis E (2015). Mid-Holocene drying of the U.S. Great Basin recorded in Nevada speleothems. Quat. Sci. Rev..

[63] Deplazes G (2013). Links between tropical rainfall and North Atlantic climate during the last glacial period. Nat. Geosci..

[64] Lachniet MS, Asmerom Y, Bernal JP, Polyak VJ, Vazquez-Selem L (2013). Orbital pacing and ocean circulation-induced collapses of the Mesoamerican monsoon over the past 22,000 y. Proc. Natl. Acad. Sci..

[65] McManus JF, Francois R, Gherardi J-M, Keigwin LD, Brown-Leger S (2004). Collapse and rapid resumption of Atlantic meridional circulation linked to deglacial climate changes. Nature.

[66] Hagos S, Leung LR, Yang Q, Zhao C, Lu J (2015). Resolution and Dynamical Core Dependence of Atmospheric River Frequency in Global Model Simulations. J. Clim..

[67] Hatchett, B. J. *et al*. Sensitivity of a western Great Basin terminal lake to winter northeast Pacific storm track activity and moisture transport. In *Special Paper 536: From Saline to Freshwater: The Diversity of Western Lakes in Space and Time* **536** (Geological Society of America, 2018).

[68] Dettinger, M. Historical and Future Relations Between Large Storms and Droughts in California. *San Franc. Estuary Watershed Sci*. **14** (2016).

[69] Rutz JJ, Steenburgh WJ, Ralph FM (2015). The Inland Penetration of Atmospheric Rivers over Western North America: A Lagrangian Analysis. Mon. Weather Rev..

[70] Dyke, A. S. An outline of North American deglaciation with emphasis on central and northern Canada. *In Developments in Quaternary Sciences* **2**, 373–424 (Elsevier, 2004).

[71] Bronk Ramsey C (2009). Bayesian Analysis of Radiocarbon Dates. Radiocarbon.

[72] Reimer P (2013). IntCal13 and Marine13 Radiocarbon Age Calibration Curves 0–50,000 Years cal BP. Radiocarbon.

[73] Allison, I. S. *Geology of pluvial Lake Chewaucan, Lake County, Oregon*. (Oregon State University Press, 1982).

[74] Frest, T. J. & Johannes, E. J. Freshwater Mollusks of the Upper Klamath Drainage, Oregon. *USDI Bur. Reclam. Rep*. 402 p (1998).

[75] Pezzopane SK, Weldon RJ (1993). Tectonic role of active faulting in central Oregon. Tectonics.

[76] Grigg LD, Whitlock C (1998). Late-Glacial Vegetation and Climate Change in Western Oregon. Quat. Res..

[77] Sea DS, Whitlock C (1995). Postglacial Vegetation and Climate of the Cascade Range, Central Oregon. Quat. Res..

[78] Adams KD, Wesnousky SG (1998). Shoreline processes and the age of the Lake Lahontan highstand in the Jessup embayment, Nevada. Geol. Soc. Am. Bull..

[79] Allen BD, Anderson RY (2000). A continuous, high-resolution record of late Pleistocene climate variability from the Estancia basin, New Mexico. Geol. Soc. Am. Bull..

[80] García AF, Stokes M (2006). Late Pleistocene highstand and recession of a small, high-altitude pluvial lake, Jakes Valley, central Great Basin, USA. Quat. Res..

[81] Ibarra DE, Egger AE, Weaver KL, Harris CR, Maher K (2014). Rise and fall of late Pleistocene pluvial lakes in response to reduced evaporation and precipitation: Evidence from Lake Surprise, California. Geol. Soc. Am. Bull..

[82] Jayko, A. S. *et al*. Late Pleistocene lakes and wetlands, Panamint Valley, Inyo County, California. In *Special Paper 439: Late Cenozoic Drainage History of the Southwestern Great Basin and Lower Colorado River Region: Geologic and Biotic Perspectives* **439**, 151–184 (Geological Society of America, 2008).

[83] Kowler, A. L. Lake Shoreline Evidence of Hydrologic Conditions in the Southern Basin and Range Province During the Late Pleistocene and Early Holocene: Paleoclimatic and Archaeological Implications. In *Human Environment Interactions* - Volume 2 1–27 (Springer Berlin Heidelberg, 2014).

[84] Kurth G, Phillips FM, Reheis MC, Redwine JL, Paces JB (2011). Cosmogenic nuclide and uranium-series dating of old, high shorelines in the western Great Basin, USA. Geol. Soc. Am. Bull..

[85] Oviatt CG (2015). Chronology of Lake Bonneville, 30,000 to 10,000 yr B.P. Quat. Sci. Rev..

[86] Smith GM, Felling DC, Wriston TA, Pattee DD (2015). The Surface Paleoindian Record of Northern Warner Valley, Oregon, and Its Bearing on the Temporal and Cultural Separation of Clovis and Western Stemmed Points in the Northern Great Basin. PaleoAmerica.

[87] Street JH, Anderson RS, Paytan A (2012). An organic geochemical record of Sierra Nevada climate since the LGM from Swamp Lake, Yosemite. Quat. Sci. Rev..

[88] Wallace JM, Lim G-H, Blackmon ML (1988). Relationship between cyclone tracks, anticyclone tracks and baroclinic waveguides. J. Atmospheric Sci..

[89] Kalnay E (1996). The NCEP/NCAR 40-Year Reanalysis Project. Bull. Am. Meteorol. Soc..

[90] Gesch D (2002). The National Elevation Dataset. Photogramm. Eng. Remote Sens..

