# Supplementary material for: North-south dipole in winter hydroclimate in the western United States during the last deglaciation
Source: Sci Rep. 2019 Mar 18;9:4826. doi: 10.1038/s41598-019-41197-y (PMC6423015; doi:10.1038/s41598-019-41197-y)
Supplement: Supplementary file 1 — Supplemental Information [file 41598_2019_41197_MOESM1_ESM.pdf]

Supplementary Information for:

North-south dipole in winter hydroclimate in the western United States during the last deglaciation

Adam M. Hudson*[1]
Benjamin J. Hatchett[2,3]
Jay Quade[4]
Douglas P. Boyle[5]
Scott D. Bassett[5]
Guleed Ali[6]
Marie G. De los Santos[7]
corresponding author, correspondence to ahudson@usgs.gov
[1]U.S. Geological Survey, Denver, Colorado, USA
[2]Division of Atmospheric Sciences, Desert Research Institute, Reno, Nevada, USA
[3]Western Regional Climate Center, Reno, Nevada, USA
[4]Department of Geosciences, University of Arizona, Tucson, Arizona, USA
[5]Department of Geography, University of Nevada-Reno, Reno, Nevada, USA
[6]Lamont-Doherty Earth Observatory, Columbia University, Palisades, New York, USA
[7]AECOM Technical Services Inc., Minneapolis, Minnesota, USA

This document contains supplementary information to support the results and interpretations of the Lake Chewaucan lake record in "North-south dipole in winter hydroclimate in the western United States during the last deglaciation." First, it includes more information on the hydrographic configuration of the Chewaucan drainage system, and a more detailed description of the samples and stratigraphic sections used to produce and interpret the lake level reconstruction. Second, we discuss the rationale for omitting an often-cited shell ^{14}C age [1](Allison, 1982), due to evidence that its elevation is inaccurate, and it does not represent a true constraint on Chewaucan lake level.

Supporting data for the Chewaucan lake level history

Basin hypsometry and hydrography

The Chewaucan lake system has four sub-basins with low gradient floors at differing elevation. These are the Summer Lake (1267 m asl), Upper Chewaucan Marsh (1315 m asl), Lower Chewaucan Marsh (1306 m asl), and Abert Lake (1294 m asl) sub-basins (Figs. S1, S2). These are divided into two hydrographic units, consisting under modern conditions of the isolated Summer Lake basin, and the hydrographically contiguous Chewaucan River basin, which flows through the Chewaucan marshes and terminates in Abert Lake (hereafter Abert Lake sub-basin). The modern Chewaucan River enters the Upper Chewaucan marsh near the low sill (1338 m asl) that separates it from the Summer Lake basin (Fig. S2). This sill is dominantly composed of alluvial fan and lacustrine gravels, and the modern river is significantly entrenched into it on the eastern side, holding the river to its current course. Samples (Fig. S3)

were collected from outcrops and stratigraphic sections in both sub-basins, and their ages were tied to lake level using the sample elevations measured by a high-precision GPS.

Stratigraphic section descriptions and interpretations

Eight stratigraphic sections from the Abert sub-basin with *in situ* ^{14}C ages on mollusk shells (Fig. S3c, d) and tufas (Fig. S3a, b, e, f) were used in the shoreline record (Fig. S4). Two sections from Licciardi (2001) were measured and resampled, and are presented here with both previous and new ^{14}C ages. The locations of each section are given in Table S2 and shown on Figure S1.

CHL13-11: CHL13-11 is located on a prominent east-facing shoreline in the northern Abert sub-basin at 1322 m asl. This section consists of five units totaling 81 cm in combined thickness. The basal unit consists of massive sandy silt with dispersed pebbles, with a vesicular pedogenic texture, and an erosional upper contact. We interpret this unit as eolian reworked sediment overprinted by pedogenesis, representing lake level below this elevation. The overlying unit consists of loose, imbricated, subrounded sandy gravel, with a sharp upper contact. We interpret it as a pebble beach deposit representing lake level at this elevation. The next overlying unit is thickest in the section—consisting of massive, fining-upward, medium-to-fine sand with an erosive upper contact. This unit contains a horizontal bed of mollusk shells ~2 cm thick that we sampled for dating. A *V. effusa* shell from this bed yielded an age of 13,670±100 cal yr BP (CHL13-11a), while a *V. effusa* shell dispersed within the upper part of this unit yielded an age of 13,390±90 cal yr BP (CHL13-11b). We interpret this unit as littoral lake sand representing lake level significantly above this elevation. The next overlying unit consists of massive

medium sand with dispersed pebbles and mollusk shells, with a gradational upper contact. A *V. effusa* shell from this unit yields an age of 13,460±100 cal yr BP (CHL13-11c), out of stratigraphic order relative to the ages below. We interpret this unit to represent eolian/colluvial reworking of the underlying lake sediment, representing lake level below this elevation. The uppermost unit is similar in sedimentary character to that of the adjacent underlying unit, but has a vesicular texture and higher clay content. It represents pedogenic development within the underlying eolian/colluvial layer.

CHL13-12: This section is located on the tread of a prominent shoreline bench on the east side of the Sawed Horn hill at an elevation of 1346 m asl. It consists of three units of 90 cm combined thickness. The basal unit consists of loose, imbricated, subrounded sandy gravel, with a sharp upper contact. We interpret this unit as a pebble shoreface beach deposit representing lake level at this elevation. The overlying unit consists of massive, well-sorted medium sand with dispersed mollusk shells throughout. It has an erosional upper contact with numerous mollusk shells directly at the upper boundary. A *V. effusa* shell from the lower part of this unit yielded an age of 14,360±300 cal yr BP (CHL13-12a). A *Pyrgylopsis* sp. shell sampled at the upper contact yielded an age of 13900±180 cal yr BP (CHL13-12b). We interpret this unit to be deposited in a backbar pool behind the prominent beach ridge located to the east, representing lake level near this elevation. The uppermost unit consists of massive, bioturbated, medium sand with pebbles and shell fragments. We interpret this unit as reworked material from the unit below mixed with colluvium and overprinted by pedogenesis, representing dry conditions and lake level below this elevation.

CHL13-13: This section is located in the bottom of a southeast-facing embayment that slopes gently towards the modern Abert Lake playa at an elevation of 1314 m asl. The section consists of three units totaling 80 cm in combined thickness. The basal unit consists of weakly calcareous laminated green silt with a sharp upper contact. We interpret it as offshore lake sediments representing lake level significantly above this location. The middle unit consists of massive medium sand with an irregular upper contact. We interpret it as beach sand representing lake level near this elevation. The upper unit consists of loose massive fine sand and contains a 4 cm thick shell layer near the base. A *V.* effusa shell from this layer yielded an age of 13,920±120 cal yr BP (CHL13-13a). This unit is interpreted as littoral sand representing lake level significantly above this elevation. The uppermost portion of the section is silty with a vesicular texture. We interpret this unit as eolian/soil modified sediment from the underlying unit representing dry conditions with lake level below this elevation.

CHL13-14: This section is located on a west-facing slope below a prominent shoreline ridge in the northern Abert Lake basin at 1326 m asl. The section consists of four units totaling 100 cm in combined thickness. The lowermost unit consists of massive fine sand coarsening to coarse sand at the top, with a gradational upper contact. The unit contains a 4 cm thick shell-rich layer near the top. A *V. effusa* shell from this layer yielded an age of 12,930±180 cal yr BP (CHL13-14-22). We interpret this unit as littoral sand transitioning to beach sand representing lake level above this elevation, but lowering. It is overlain by interbedded gravel and sand with dispersed mollusk shells and shell fragments and an erosive upper contact. A *V. effusa* shell from this unit yielded an age of 13,620±140 cal yr BP (CHL13-14-40), out of stratigraphic order. Coupled with the abundant shell fragments in this unit, this shell was likely reworked from an older unit. We

interpret this unit as beach sand and gravel representing lake level at this elevation. It is overlain by massive, poorly sorted sandy gravel with angular clasts. We interpret this unit as colluvium representing lake level below this elevation. The uppermost unit consists of massive fine sand with extensive modern root bioturbation. It is interpreted as eolian sand representing dry conditions and lake level significantly below this elevation.

CHL13-16: This section is located on a prominent shoreline ridge on the northernmost side of the Abert Lake basin at 1324 m asl. It consists of nine units totaling 400 cm in combined thickness. The lowermost unit consists of imbricated, bedded, well-rounded gravel with an erosional upper contact. We interpret this unit as beach gravel, representing lake level at this elevation. The overlying unit is cross-bedded coarse sand with pebble layers, with reworked shells at the base of the unit and an inclined, sharp upper contact. A *V. effusa* shell from this location yielded an age of 13,760±190 cal yr BP (CHL13-16b). We interpret this unit as shoreface sands representing lake level slightly above this elevation. The next overlying unit consists of imbricated, bedded, well-rounded gravel with dispersed shells and a sharp upper contact. A *Pyrgylopsis* sp. shell from the middle of this unit yields an age of 14,690±420 cal yr BP (CHL13-16c), out of stratigraphic order and is likely reworked from an older unit. We interpret this unit as beach gravel representing lake level at this elevation. The next overlying unit consists of massive medium sand with large angular gravel dispersed throughout and an erosional upper contact. We interpret this unit as eolian sand and colluvium representing lake level below this elevation. The next overlying unit consists of beach gravel similar to the units below. It is overlain by shoreface sands similar to those below, an additional beach gravel unit,

and is capped at the top by a similar eolian sand and colluvium layer with pedogenic development.

CHL13-19: This section is located in a transverse roadcut through a prominent shoreline bench near the modern Abert Lake elevation on the northeastern lake margin. It is at an elevation of 1302 m asl at the base and up to 1308 m asl near the elevation of the shoreline terrace tread at ~1310 m asl. The section consists of six units totaling 660 cm in combined thickness. The basal unit consists of weakly bedded, well-sorted medium sand with a gradational upper contact. We interpret this unit as littoral sand representing lake level above this elevation. The overlying unit consists of well-sorted, sub-rounded pebble gravel grading upward to moderately sorted cobble gravel with a sharp upper contact. The gravel unit contains tufa-coated clasts, increasing in frequency towards the top of the unit. Tufa from an *in situ* clast in the upper part of the unit yielded an age of 21,240±250 cal yr BP (CHL13-19a). We interpret this unit as shoreface grading upward to beach gravel representing lake level near or at this elevation. The next overlying unit consists of well-bedded, well-sorted medium sand grading upward to fine sand with a sharp upper contact and a layer of mollusk shells at the contact. A *V. effusa* shell from this location yielded an age of 13,615±130 cal yr BP (CHL13-19b). We interpret this unit as littoral grading to offshore sand representing lake level increasingly above this elevation. The next overlying unit consists of massive, well-sorted coarse sand with an erosional upper contact. We interpret this unit as littoral sand representing lake level above this elevation. The next overlying unit consists of well-sorted, sub-rounded pebble gravel grading upward to moderately sorted cobble gravel with a sharp upper contact. The gravel unit contains tufa-coated clasts, increasing in frequency towards the top of the unit. Tufa from an *in situ* clast in the upper part of

the unit yielded an age of 10,840±240 cal yr BP (CHL13-19c). A *V. effusa* shell from this location yielded an age of 13,750±190 cal yr BP (CHL13-19d), and likely was reworked into this deposit from the shell bed in the units below. We interpret this unit as shoreface grading upward to beach gravel representing lake level near or at this elevation. The uppermost unit consists of massive silty sand with a vesicular texture. We interpret this unit as eolian sediment representing dry conditions and lake level below this elevation.

CHL14-32: This section is located on the extreme southeastern shoreline of Abert Lake in a transverse roadcut through the same prominent shoreline riser as described in CHL13-19 above. This section was previously described and dated (Licciardi, 2001), and was resampled and described here, incorporating previous ages. It consists of seven units totaling 800 cm in combined thickness. The basal unit (not previously described) consists of poorly exposed subangular boulder gravel with tufa coatings on the clasts and an unconformable but depositional upper contact. Tufa from an in situ clast in the upper part of the unit yielded an age of 18060±250 cal yr BP (CHL14-32-1). We interpret this unit as beach gravel and colluvial boulders. The overlying unit (Licciardi's Qps) consists of interbedded coarse sand and pebble layers with steep lakeward-dipping lenticular beds and occasional silt layers with sharp upper contact. We interpret this unit as proximal deltaic sediment issuing from a steep drainage on the Abert Rim creating a fan-shaped headland of sediment grading to the modern Abert playa to the north of the section. The next overlying unit (Licciardi's Qss) consists of interbedded lakeward-dipping laminae of silt and fine sand with an erosional upper contact. We interpret this unit as distal deltaic sediment representing lake level significantly above this elevation. The next overlying unit (Licciardi's Qg1) is loose and poorly exposed, consisting of poorly sorted rounded

cobbles and sub-angular boulders with tufa coats. Tufa from an *in situ* clast in the upper part of the unit yielded an age of 14,500±360 cal yr BP (CHL13-32-4). It is interpreted as beach gravel mixed with colluvial boulders representing lake level at this elevation. The next overlying unit (Licciardi's Qs) is also poorly exposed, consisting of massive coarse sand with dispersed shells and a layer of shells and shell fragments near the erosional upper contact. A *V. effusa* shell from the middle of this unit yielded an age of 13,410±290 cal yr BP (sample 7EG of Licciardi, 2001), whereas a *V. effusa* shell from the upper shell layer yielded an age of 13,720±230 cal yr BP (CHL14-32-5, this study). We interpret this unit as littoral sand representing lake level above this elevation. The uppermost unit consists of a rounded cobble gravel coarsening upward to a subangular boulder gravel with tufa coatings on clasts throughout and dispersed shells. A *V. effusa* shell from the lower part of this unit yielded an age of 13,880±250 cal yr BP (sample 7FG of Licciardi, 2001) and was likely reworked from the unit below. Tufa from an *in situ* clast in the upper part of the unit yields an age of 11020±210 cal yr BP (CHL13-32-3a, this study).

CHL15-56: This section is located in a transverse roadcut through the same shoreline terrace and between the previous two sections along the eastern side of Abert Lake. It consists of four units totaling 320 cm in combined thickness. It is the same section as Section 6 of Licciardi (2001) and previous ages are incorporated here. The basal unit consists of massive silt with an erosional upper contact. We interpret this unit as offshore lake silt representing lake level significantly above the section elevation. The overlying unit consists of massive medium sand with a 6 cm-thick mollusk shell bed near the erosional upper contact (Fig. S3c). One *V. effusa* shell from this bed yielded an age of 13,520±200 cal yr BP (sample 6AV of Licciardi, 2001), while a second yielded an age of 13,880±170 cal yr BP (CHL15-56-1, this study). We interpret this unit as

littoral sand representing lake level above this elevation. The next overlying unit consists of rounded cobble coarsening upward to sub-angular boulder gravel with dispersed shell fragments and tufa coatings on clasts. Tufa from an *in situ* clast in the upper part of the unit yielded an age of 11,640±300 cal yr BP (CHL15-56-2, this study). We interpret this unit as beach gravel mixed with colluvial boulders representing lake level at this elevation. The uppermost unit consists of massive root-bioturbated fine sand. We interpret this unit as eolian sediment representing dry conditions and lake level lower than this elevation.

Synthesis of stratigraphy, and detailed constraints on lake level highstands:

This section provides more detail on specific samples and stratigraphic relationships that were used to infer the range of lake levels represented during the LGM (>18.0 ka), B/A (14.5-12.9 ka), and early Holocene (11.5-9.5 ka) lake stands. Because this lake system remained in a continually closed-basin state throughout the Deglacial period, lake level likely varied significantly in response to multi-decadal and centennial-scale hydroclimate variability that is not clearly resolvable within the dating resolution of ^{14}C ages on discontinuous shore facies sediments. Thus, we have assigned conservatively wide ranges in lake elevation for each of these millennial-scale lake stand intervals based on the range in sample elevations and correlative stratigraphic units deposited during each (Fig. S2). In addition to climate variability, Pezzopane and Weldon (1993) estimated significant recent offset on active normal faults within both sub-basins of several meters, sufficient to induce additional change to sample and sediment elevations. Because the total change in lake level indicated by these samples is >50 m, and we interpret lake level changes only at the millennial scale, these location and age uncertainties do not significantly affect the paleoclimate interpretations of our work.

LGM lowstand: Tufa ages and stratigraphic evidence from both sub-basins constrain the lake level of Abert and Summer Lakes during the LGM period. A pair of lake lowstands, occurring at ~21.0 ka and ~18.0 ka, were constrained in the Abert Lake sub-basin by several dates on tufas on >1 m diameter boulders located just above the historical shoreline level along the Abert Rim. These boulders were initially deposited by rock fall or debris flows from the Abert Rim and have remained relatively static since, winnowed and incorporated into the shoreline deposits. Tufa was deposited on them during the LGM lowstand; they were subsequently buried by littoral sediments during the highstands; and they were exhumed by wave action during the lowstand of the late Holocene. Previous burial in highstand lake sediments is corroborated by LGM tufa ages (CHL13-19a; CHL14-32-1) obtained from shoreline gravels buried beneath deep lake sediments in sections CHL13-19 and CHL14-32 where the fine-grained units are armored by the overlying early Holocene shoreline gravels (Fig. S4). In the Summer Lake sub-basin, two tufa ages collected from a shoreline in the northeastern area yielded ages of 17.8 and 19.3 ka, respectively. This suggests Summer Lake was deeper and had a proportionally greater surface area during the LGM period (21.3-17.9 ka) relative to Abert Lake (Fig. S2). This difference in lake level is difficult to explain with the modern hydrographic configuration, because the Abert Lake drainage basin is much larger, and has a much greater area of high elevation terrain. Under this condition, we would expect Abert Lake to fill more quickly and attain greater steady-state lake area relative to Summer Lake, the opposite of what is observed during the LGM. Previous researchers have suggested that prior to the Deglacial basin-integrating highstand, the Chewaucan River may have flowed northwest across the sill into the Summer Lake basin where the river exits the mountain front (Allison, 1982; Davis, 1985). Our shoreline reconstruction

supports this conclusion. It is noteworthy, and particularly important for future studies trying to quantify past precipitation changes through hydrologic budget modeling. However, it makes little difference for the conclusions presented in this paper, as the LGM lakes were still much smaller than the B/A basin-integrating highstand, and there is no evidence in either hydrographic basin for an HS1 or YD lake.

B/A highstand: The majority of materials collected in both sub-basins of the Chewaucan lake system were deposited during this highstand period. In the Abert Lake sub-basin, tufas of ~14.5 ka age have been found at elevations ranging from 1308-1350 m asl, all but one (CHL15-32-4) occurring >1340 m asl. In the northern Summer Lake sub-basin, similar ages were obtained by Friedel (2001) in the region of Klippel Point (Fig. S1) at 1310-1337 m asl, below the spilling threshold. We obtained a similar age (CHL15-85 base) from the high shoreline complex (1345 m asl) to the east in the Sheeplick Draw area. These ages are all indistinguishable from each other within error (Table S2). We regard the best explanation for the range of elevations represented by these overlapping ages to be a relatively rapid rise in lake level, on the order of a century, to a steady-state highstand coincident with the onset of the Bølling warming. Several shell ages of ~14.5 ka also fall at the highstand elevation (e.g., CHL13-12a), or are reworked into younger deposits (e.g., CHL13-16c; ZX-1-1) at lower elevation, providing further evidence that a significant lake was present at this time.

Between 13.4-14.0 ka, 15 ages on shell and 11 on tufa support a continuation of deep lake conditions. The shells have been spread in elevation by significant reworking from the high shoreline, creating the conspicuous shell bed which crops out widely in the Abert sub-basin. All tufas sampled in the Abert sub-basin of this age are above the spilling threshold, while those

from the Summer sub-basin vary between the spilling threshold and ~20 m below (Fig. S2), suggesting Summer Lake may have at times fallen below the threshold, and was maintained by overflow from the Abert sub-basin. There is a clear overflow channel from the Upper Chewaucan Marsh to Summer Lake visible on satellite imagery (Fig. S5), which further supports this interpretation. Finally, two ages (CHL13-14-22, CHL15-87 top) indicate water level retreated below the spilling threshold in both basins at ~12.9 ka. After this, no ages were obtained until those dating to the early Holocene in the Abert sub-basin.

Early Holocene mid-stand, late Holocene lowstand: Eight ages, all on tufa from the Abert sub-basin, indicate a significant lake stand was achieved during the early Holocene, 11.5-9.5 ka. The highest elevation samples were found at ~1314 m asl, which would have created a lake filling the Upper and Lower Chewaucan Marshes, but still well below the spilling threshold to the Summer sub-basin (Fig. S1, S2). No shells of this age range were found, suggesting the lake was too saline to support mollusks. No early Holocene ages were obtained from the Summer Lake sub-basin either, in line with the hypothesis that the Chewaucan River was diverted into the Abert sub-basin following the highstand (Allison, 1982; Davis, 1985), isolating Summer Lake from the primary runoff-producing area of the basin. Finally, seven ages, all on tufa from the modern playa edge in the Abert sub-basin, indicate Abert Lake fluctuated at low lake level within the range of modern variability (~1295-1300 m asl) since 3.9 ka.

The high shoreline age obtained by Allison (1982): Prior to recent detailed sampling in this study and Egger et al. (2018), only one age was published from the integrated highstand elevation of Lake Chewaucan at ~1350 m asl (Allison, 1982). This age of 17,500±300 ^{14}C yr (21.2 ka; Table

S2), obtained on multiple shells of unknown taxa, has been often cited as evidence for highstand lake conditions for Lake Chewaucan during the LGM interval (Ibarra et al., 2014; Licciardi, 2001; Lyle et al., 2012; Smith et al., 2015). However, this age is inconsistent with our evidence from the Abert sub-basin, which places lake level near the modern playa at 21.2 ka (sample CHL-19a, Table S2). It is also inconsistent with the sediment characteristics of the uppermost sediments from the northern Summer Lake basin exposed in the Ana River outcrops and two Summer Lake cores, which suggest only moderate lake conditions during the LGM period (Cohen et al., 2000). Lastly, it is inconsistent with compilations of paleoclimate records from the surrounding region of the Pacific Northwest, which indicate relatively dry, cold conditions during the LGM period (Lora et al., 2017; Oster et al., 2015a).

Allison (1982) lists the location for the shell sample, collected in 1979, as "NW1/4 Sec. 34, T. 31 S., R. 18 E., near Ten Mile Butte." He lists the elevation as 4425 ft (1349 m asl), based on a barometric Paulin aneroid altimeter measurement calibrated to a benchmark three miles (~5 km) away. However, using the 10 m digital elevation model presented in Figure S1 (Gesch et al., 2002), there is no location within that quarter section (red square in Figure S1, S6) with an elevation less than 1362 m asl. Therefore, the quoted elevation cannot be correct, using the datum of this DEM. This quarter section is also located in the drainage of a small internally drained playa within the Chewaucan drainage system with a minimum elevation of 1361 m asl (Fig. S6). If this location is correct, then this sample cannot be collected at 1349 m asl, and does not directly constrain the lake elevation of a Lake Chewaucan shoreline. Based on this evidence, we have omitted this age from our record.

Supplemental References

Allison, I.S., 1982. Geology of pluvial Lake Chewaucan, Lake County, Oregon, Oregon State monographs. Oregon State University Press, Corvallis, Or.

Cohen, A., Palacios-Fest, M., Negrini, R., Wigand, P., Erbes, D., 2000. A paleoclimate record for the past 250,000 years from Summer Lake, Oregon, USA: II. Sedimentology, paleontology and geochemistry. J. Paleolimnol. 24, 151–182. https://doi.org/10.1023/A:1008165326401

Davis, J.O., 1985. Correlation of late Quaternary tephra layers in a long pluvial sequence near Summer Lake, Oregon. Quat. Res. 23, 38–53. https://doi.org/10.1016/0033-5894(85)90070-5

Egger, A.E., Ibarra, D.E., Weldon, R.J., Langridge, R., Marion, B., Hall, J., 2018. Influence of pluvial lake cycles on earthquake recurrence in the northwestern Basin and Range, USA. Geol. Soc. Am. Spec. Pap. 536. https://doi.org/10.1130/2018.2536(07)

Friedel, D., 2001. Pleistocene Lake Chewaucan: two short pieces on hydrological connections and lake-level oscillations, in: Quaternary Studies near Summer Lake, Oregon, Friends of the Pleistocene Field Trip Guide.

Gesch, D., Oimoen, M., Greenlee, S., Nelson, C., Steuk, M., Tyler, D., 2002. The National Elevation Dataset. Photogramm. Eng. Remote Sens. 68, 5–11.

Ibarra, D.E., Egger, A.E., Weaver, K.L., Harris, C.R., Maher, K., 2014. Rise and fall of late Pleistocene pluvial lakes in response to reduced evaporation and precipitation: Evidence from Lake Surprise, California. Geol. Soc. Am. Bull. 126, 1387–1415. https://doi.org/10.1130/B31014.1

Licciardi, J.M., 2001. Chronology of latest Pleistocene lake-level fluctuations in the pluvial Lake Chewaucan basin, Oregon, USA. J. Quat. Sci. 16, 545–553. https://doi.org/10.1002/jqs.619

Lora, J.M., Mitchell, J.L., Risi, C., Tripati, A.E., 2017. North Pacific atmospheric rivers and their influence on western North America at the Last Glacial Maximum. Geophys. Res. Lett. 44. https://doi.org/10.1002/2016GL071541

Lyle, M., Heusser, L., Ravelo, C., Yamamoto, M., Barron, J., Diffenbaugh, N.S., Herbert, T., Andreasen, D., 2012. Out of the Tropics: The Pacific, Great Basin Lakes, and Late Pleistocene Water Cycle in the Western United States. Science 337, 1629–1633. https://doi.org/10.1126/science.1218390

Oster, J.L., Ibarra, D.E., Winnick, M.J., Maher, K., 2015. Steering of westerly storms over western North America at the Last Glacial Maximum. Nat. Geosci. 8, 201–205. https://doi.org/10.1038/ngeo2365

Smith, G.M., Felling, D.C., Wriston, T.A., Pattee, D.D., 2015. The Surface Paleoindian Record of Northern Warner Valley, Oregon, and Its Bearing on the Temporal and Cultural Separation of Clovis and Western Stemmed Points in the Northern Great Basin. PaleoAmerica 1, 360–373. https://doi.org/10.1179/2055557115Y.0000000001

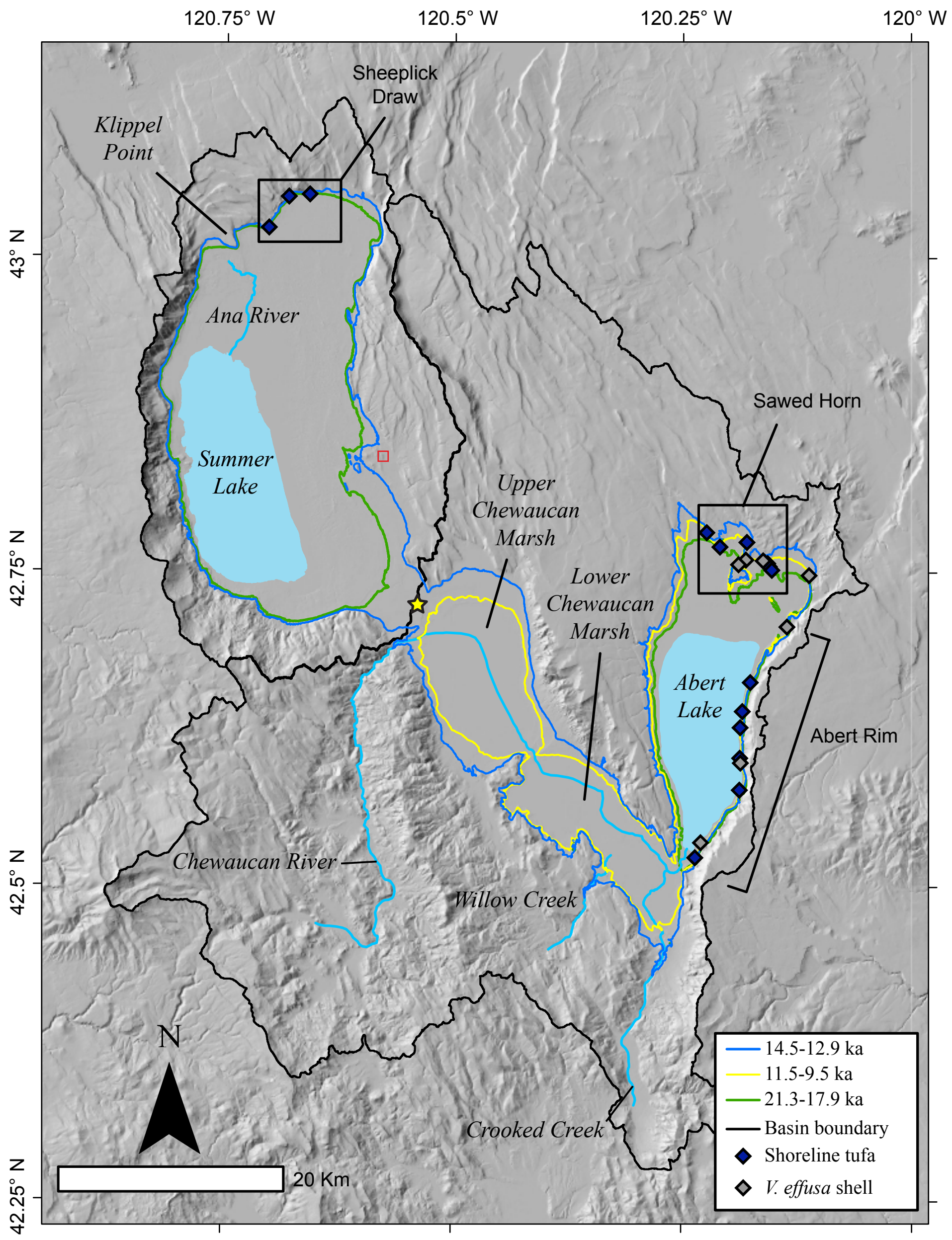

Figure S1. Lake Chewaucan basin map. Basin boundary is shown in black, the Bolling-Allerod highstand lake extent in blue, the LGM lake extents in green, and the early Holocene extent of Abert Lake in yellow. Approximate extents of modern Summer Lake and Abert Lakes shown in light blue. Sampling locations for shoreline tufas and shells from this study are shown by blue and gray diamonds, respectively. Named areas where samples were collected are labeled (e.g. the Abert Rim). Overflow point (1338 m asl) connecting the Summer Lake sub-basin and Abert Lake sub-basin shown by yellow star. Location of shell ^{14}C age sample from Allison, 1982 that was omitted from our record is shown by red box, clearly well outside the high shoreline elevation. Base hillshade was created using ArcGIS 10.4 using the 10 m-resolution digital elevation model from the USGS National Elevation Dataset (Gesch et al., 2002; available from https://nationalmap.gov).

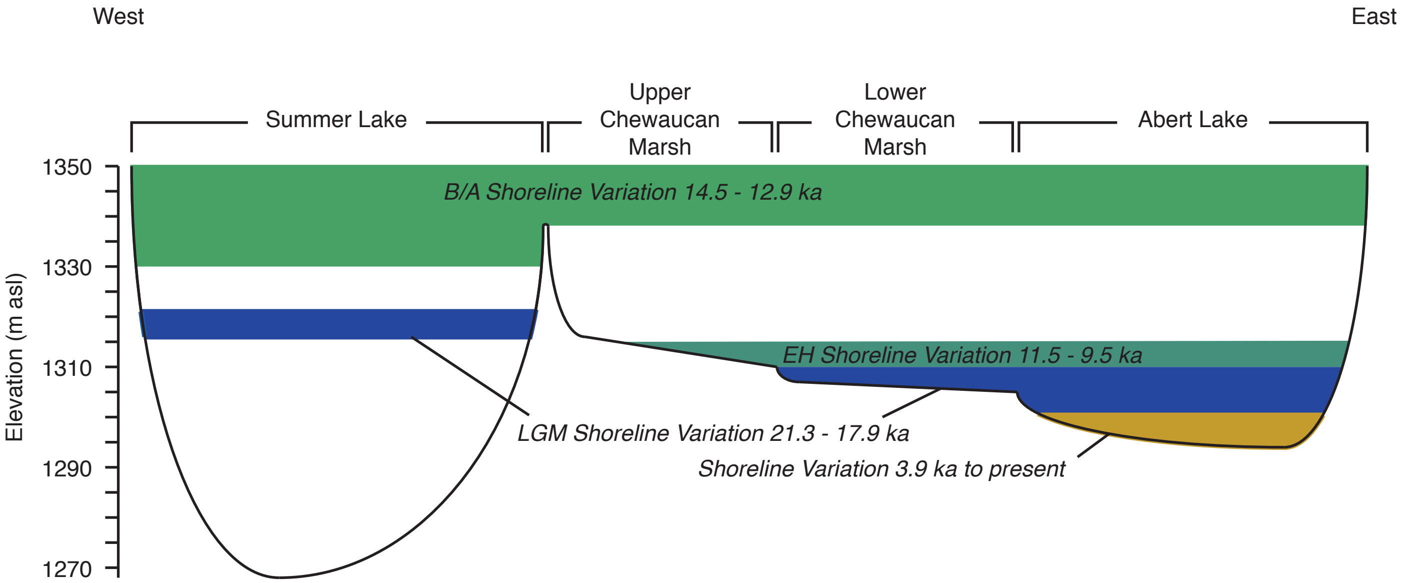

Figure S2. Schematic cross-section of Chewaucan lake system from Summer Lake to Abert Lake sub-basins. Variation in lake surface elevation during each highstand interval depicted by colored bars. Water depth at each shoreline complex is indicated by the distance between the colored bar and the lake bottom (black outline) in each sub-basin. Variation estimated by variability in shoreline and sample elevations for each age group.

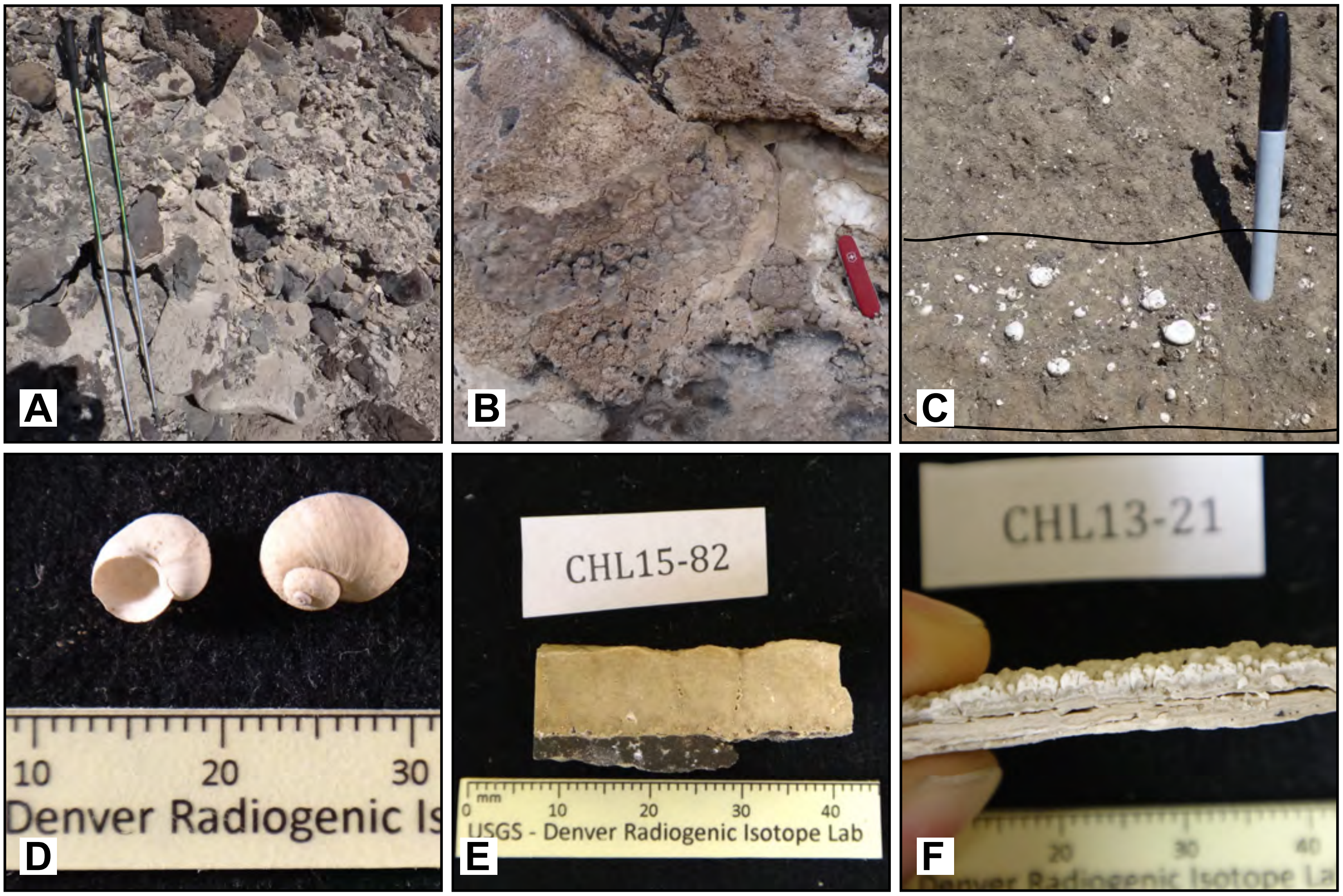

Figure S3. Field and sample photographs of Chewaucan carbonates. All photo credits: A. Hudson, September 2014 - May 2016. A - tufa-cemented high shoreline (1346-1350 m asl) gravel from Sheeplick Draw locality, north Summer Lake sub-basin. Trekking pole for scale is 130 cm in length. B - tufa cementing shoreline boulders along the Abert Rim. Pocket knife for scale is ~12 cm in length. C - Mollusk coquina deposit exposed in section CHL15-56. Pen for scale is 20 cm in length. D - Fossil *V. effusa* mollusk shells collected from littoral deposits (CHL13-14b, 1326 m asl) in the Sawed Horn locality, north Abert Lake sub-basin. Scale is in mm. E - cross-section of tufa sample (CHL15-82) from the Sheeplick Draw locality, north Summer Lake sub-basin. Sca1e is in mm. F - cross-section of tufa sample (CHL13-21, 1296 m asl) from Abert Rim locality showing lower laminated horizons overlain by upper dendritic horizons.
Scale is in mm.

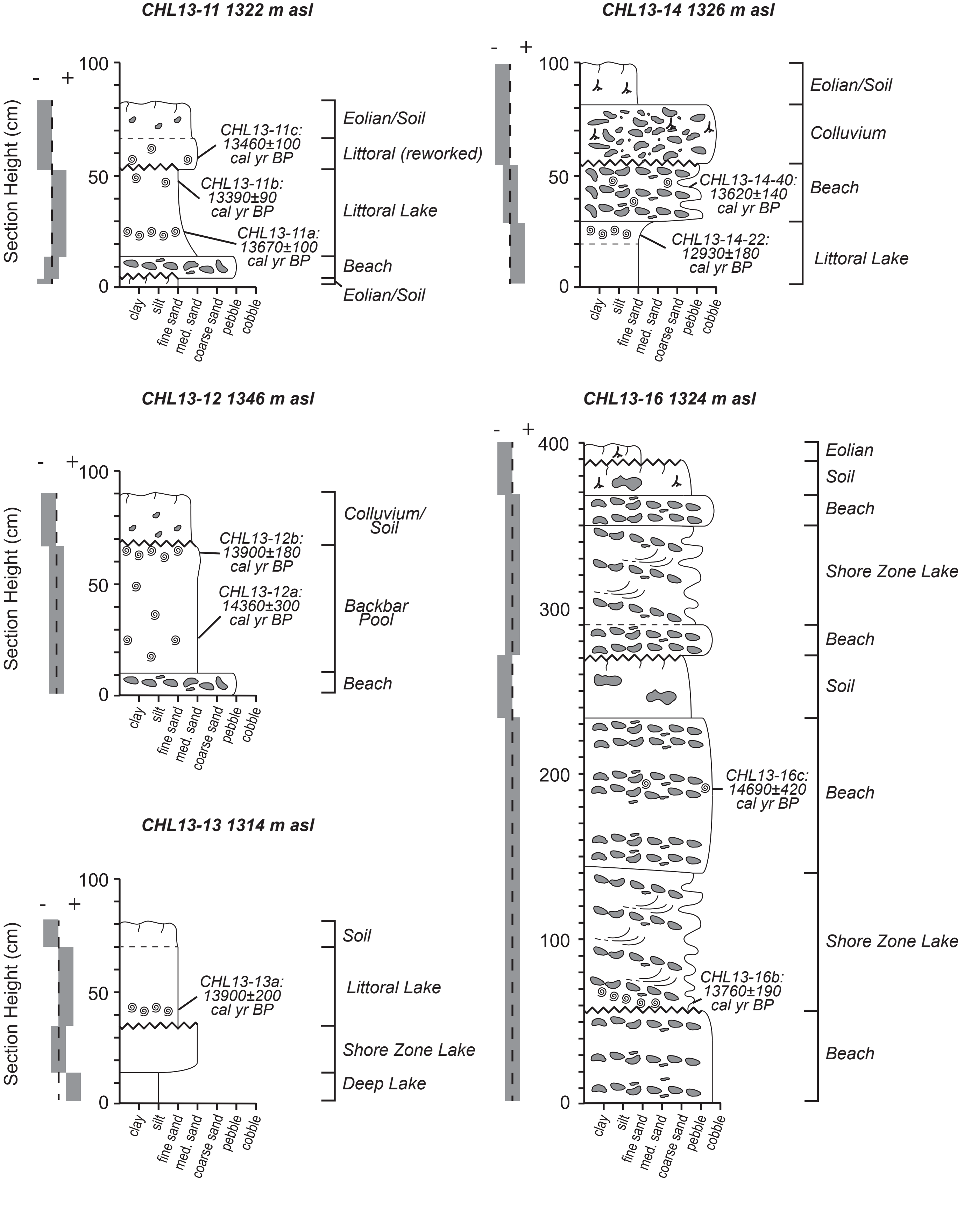
CHL13-11 1322 m asl
Section Height (cm)
- +
100
50
0
CHL13-11c:
13460±100
cal yr BP
CHL13-11b:
13390±90
cal yr BP
CHL13-11a:
13670±100
cal yr BP
Eolian/Soil
Littoral (reworked)
Littoral Lake
Beach
Eolian/Soil
clay
silt
fine sand
med. sand
coarse sand
pebble
cobble
CHL13-14 1326 m asl
CHL13-14-40:
13620±140
cal yr BP
CHL13-14-22:
12930±180
cal yr BP
Eolian/Soil
Colluvium
Beach
Littoral Lake
CHL13-12 1346 m asl
CHL13-12b:
13900±180
cal yr BP
CHL13-12a:
14360±300
cal yr BP
Colluvium/
Soil
Backbar
Pool
Beach
CHL13-13 1314 m asl
CHL13-13a:
13900±200
cal yr BP
Soil
Littoral Lake
Shore Zone Lake
Deep Lake
CHL13-16 1324 m asl
400
300
200
100
Eolian
Soil
Beach
Shore Zone Lake
Beach
Soil
CHL13-16c:
14690±420
cal yr BP
Beach
Shore Zone Lake
CHL13-16b:
13760±190
cal yr BP
Beach

Fig. S4

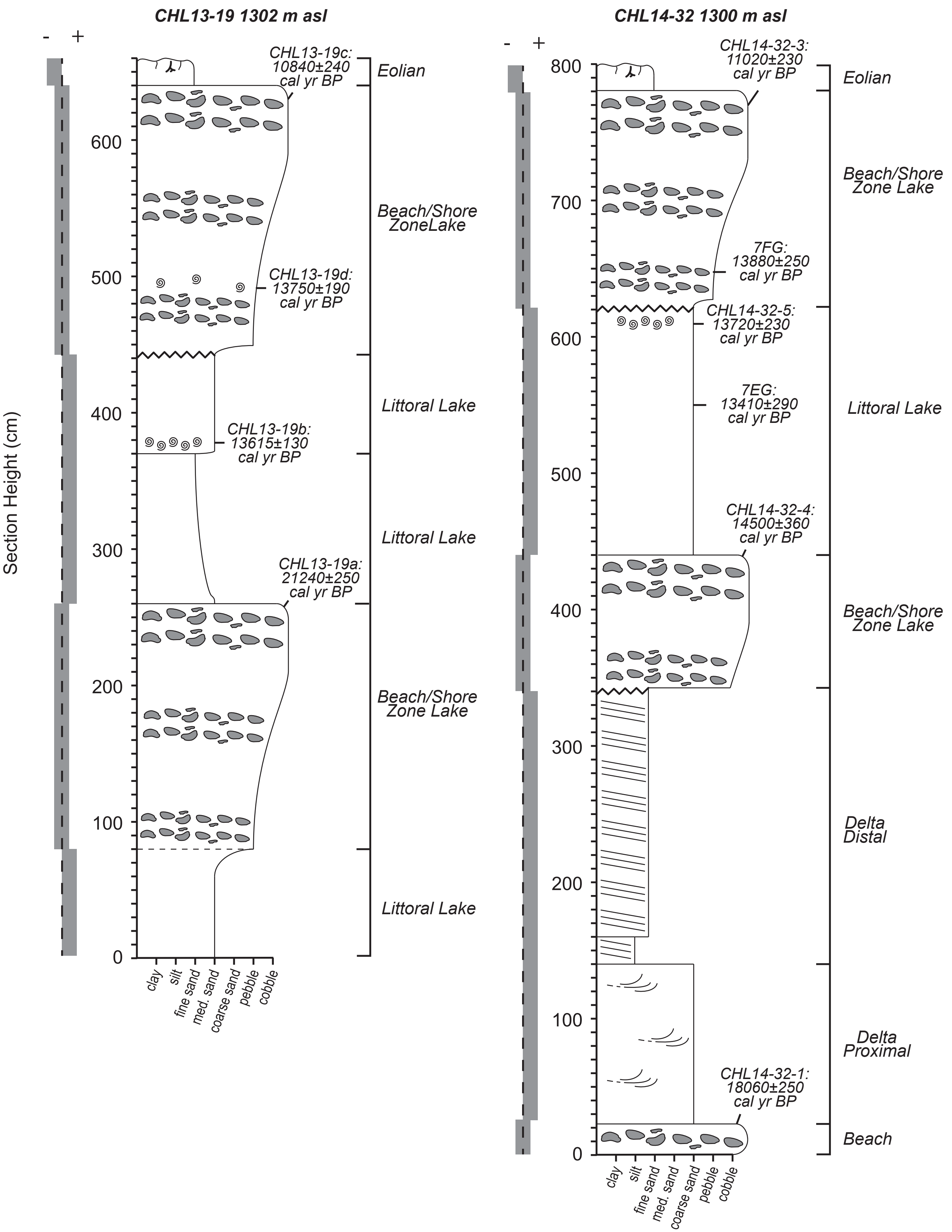
CHL13-19 1302 m asl
CHL14-32 1300 m asl
Section Height (cm)
CHL13-19c:
10840±240
cal yr BP
CHL13-19d:
13750±190
cal yr BP
CHL13-19b:
13615±130
cal yr BP
CHL13-19a:
21240±250
cal yr BP
Eolian
Beach/Shore
ZoneLake
Littoral Lake
Littoral Lake
Beach/Shore
Zone Lake
Littoral Lake
clay
silt
fine sand
med. sand
coarse sand
pebble
cobble
CHL14-32-3:
11020±230
cal yr BP
7FG:
13880±250
cal yr BP
CHL14-32-5:
13720±230
cal yr BP
7EG:
13410±290
cal yr BP
CHL14-32-4:
14500±360
cal yr BP
CHL14-32-1:
18060±250
cal yr BP
Eolian
Beach/Shore
Zone Lake
Littoral Lake
Beach/Shore
Zone Lake
Delta
Distal
Delta
Proximal
Beach

Fig. S4 contd.

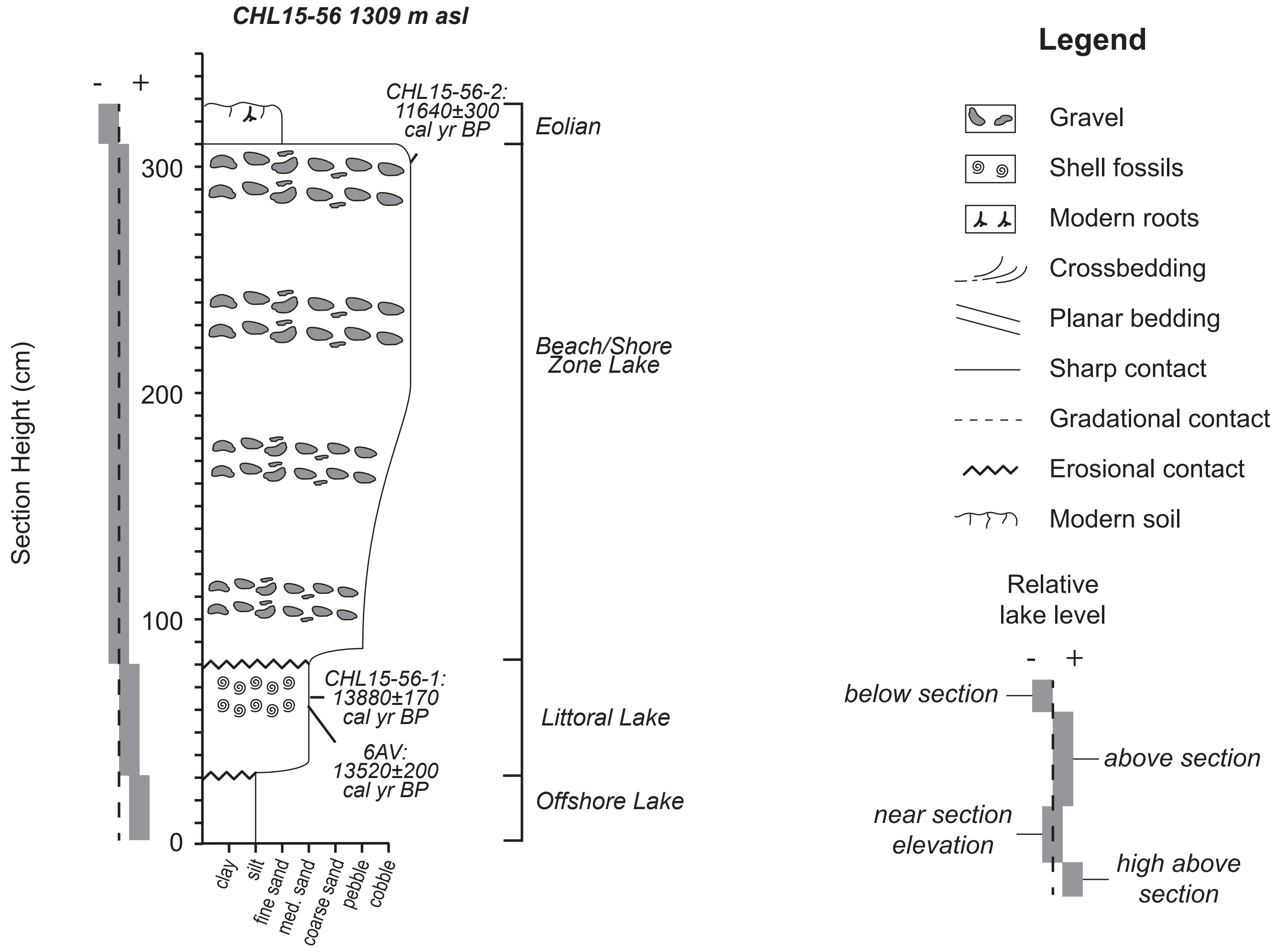

Figure S4 contd. Lake sediment stratigraphic sections and ^{14}C dating samples. Locality names and elevations are shown above each section. *In situ* ages are labeled where sampled with sample name (see Table S1 for more information) and calendar age and 2 sigma uncertainties. Depositional environment interpretations for each unit are shown by brackets to the right of each section. Lake surface elevation relative to the section for each stratigraphic unit is indicated by the relative lake level bar to the left of each section, with the position of the bar denoting lake level (gray bars) above or below the stratigraphic unit being described (dotted line).

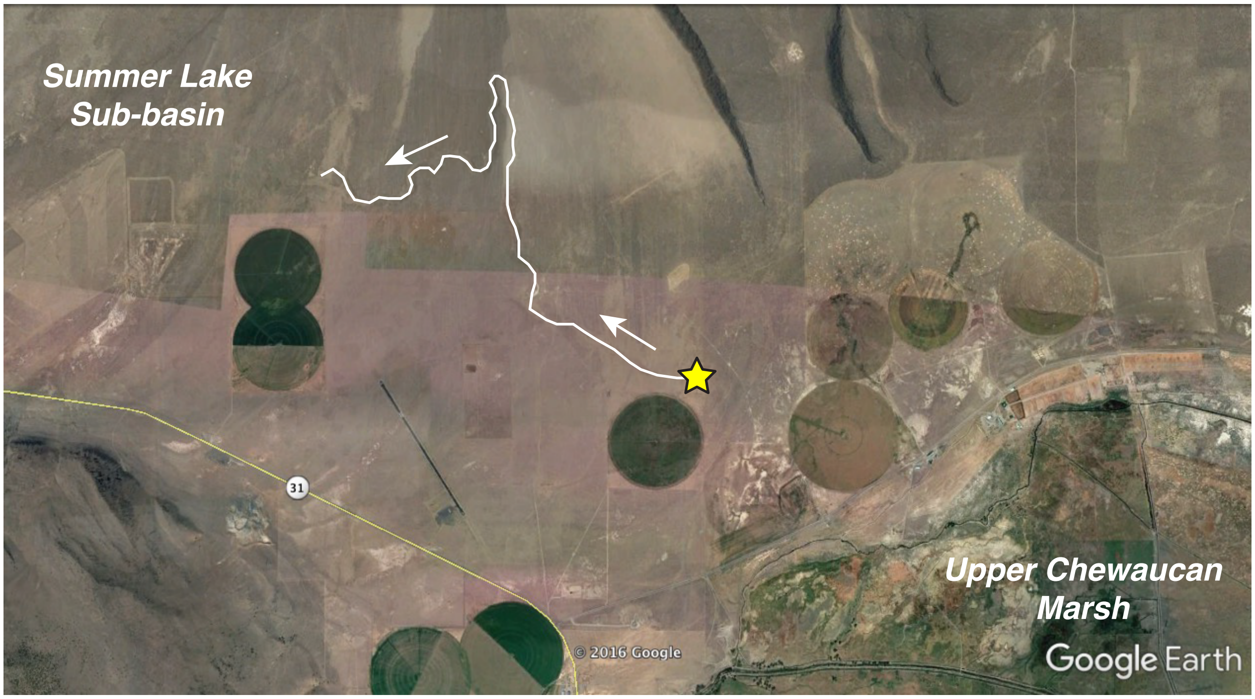

Figure S5. Google Earth image of overflow channel from Upper Chewaucan Marsh (Abert sub-basin) to the Summer Lake sub-basin. Channel shown by white line, with direction of flow indicated by white arrows. Yellow star shows drainage basin spill point, located at the same location as the yellow star in Figure S1. Map data © 2016 Google.

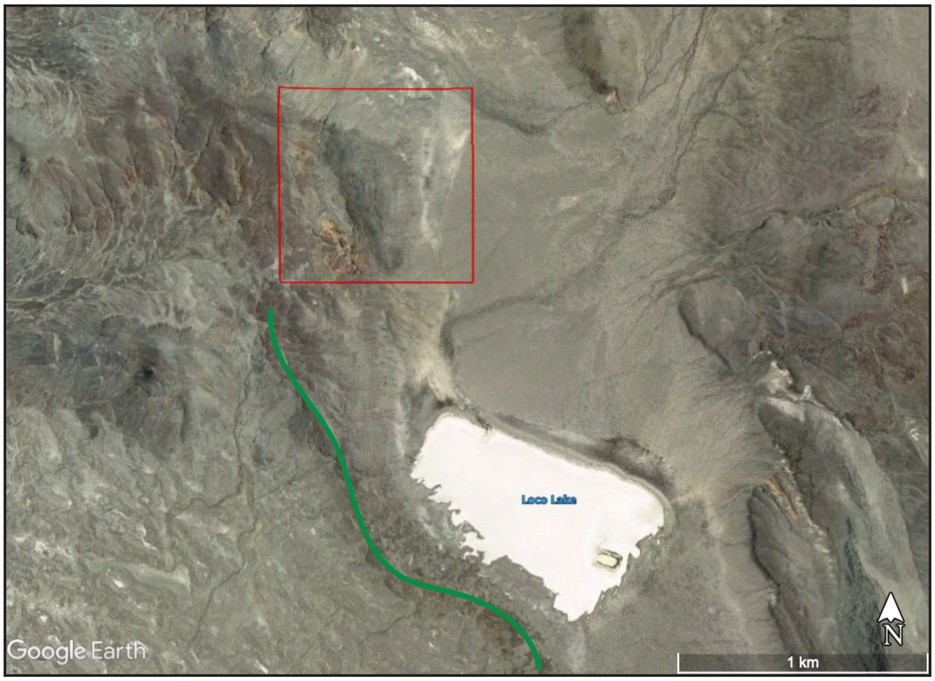

Figure S6. Allison (1982) sample location. NW 1/4, section 34, T. 31 S., R 18E. shown by red box (also shown in Figure S1). This location is mostly tributary to 'Loco Lake' playa to the south, which is separated from the Chewaucan drainage by a low divide on its west (green line). Base image map data © 2018 Google.

Table S1. Paleohydrologic and paleoclimate records used in regional compilation (shown in Figure 1a), and climate inferences for key time intervals.

Site or Record Name	Record Number on Fig. 1a	Latitude	Longitude	Elevation (m asl)	HS1b status (16.1-14.6 ka)[a]	B/A status (14.6-12.8 ka)[a]	Record Type	Record continuity[b]	Age model (age resolution at 2 sigma)	Avg 2 sigma age uncertainty (# of ages)	Reference
Indian Prairie Fen, Cascades, OR	1	44.63	-122.58	988	1	2	terrestrial pollen in bog sediments	C	^{14}C ages bulk lake sediment	480 cal yr (n = 2)	Sea and Whitlock, 1995; compiled by Briles et al., 2005
Gordon Lake, Cascades, OR	2	44.35	-122.25	1177	1	2	terrestrial pollen in lake sediments	C	^{14}C ages charcoal, bulk sediment	370 cal yr (n = 2)	Grigg and Whitlock, 1998; compiled by Briles et al., 2005
Little Lake, Coast Range, OR	3	44.17	-123.58	217	1	2	terrestrial pollen in lake sediments	C	^{14}C ages bulk lake sediment	730 cal yr (n = 8)	Grigg and Whitlock, 1998; compiled by Briles et al., 2005
Warner Lake, OR	4	42.81	-119.6	1360	0	2	lake level dating, outcrop-based	D	^{14}C ages mollusk shells	230 cal yr (n = 3)	Wriston and Smith, 2017
Caledonia Marsh, Klamath Lake, OR	5	42.4	-121.9	1265	1	2	diatom assemblage, in lake sediments	C	^{14}C ages bulk lake TOC	310 cal yr (n = 5)	Bradbury et al., 2004
Bolan Lake, Siskiyou Mtns, OR	6	42	-123.5	1638	1	2	terrestrial pollen in lake sediments	C	^{14}C ages wood, gyttja	320 cal yr (n = 2)	Briles et al., 2005
ODP 1019	7	41.7	-125	n/a	1	3	terrestrial pollen in ocean sediments	C	^{14}C ages marine forams	390 cal yr (n = 5)	Lyle et al., 2012
Lake Surprise, CA	e	41.5	-120	1400	2	2	lake level dating, outcrop-based	D	^{14}C ages tufa, U-Th ages tufa	^{14}C: 230 cal yr (n = 16)	Ibarra et al., 2014; Egger et al., 2018
Lake Bonneville, UT	9	40.5	-113	1286	2	1	lake level dating, outcrop-based	D	^{14}C ages, carbonates and organics	450 cal yr (n = 142)	Oviatt, 2015
Lake Franklin, NV	f	40.435	-115.307	1815	2	1	lake level dating, outcrop-based	D	^{14}C ages on mollusk shells	410 cal yr (n = 31)	Munroe and Laabs, 2013
Lake Lahontan, NV	g	39.8	-118.8	1180	2	1	lake level dating, outcrop-based	D	^{14}C ages on tufa, mollusk shells, bone	270 cal yr (n = 56)	Benson et al., 2013; Adams and Wesnousky, 1998
Newark Lake, NV	12	39.635	-115.726	1783	2	0	lake level dating, outcrop-based	D	^{14}C ages on mollusk shells	530 cal yr (n = 6)	Kurth et al., 2011
Jakes Lake, NV	13	39.27	-115.25	1922	2	0	lake level dating, outcrop-based	D	^{14}C ages on mollusk shells	260 cal yr (n = 3)	Garcia and Stokes, 2006
McLean's Cave, Moaning Cave, CA	14	38.07	-120.44	300	2	3	speleothem isotopes and trace elements	C	U-Th ages on speleothem	330 cal yr (n = 11)	Oster et al., 2015b
Swamp Lake, CA	15	37.95	-119.5	1554	3	3	carbon isotopes, organic content	C	^{14}C ages on bulk sediment	240 cal yr (n = 2)	Street et al., 2012
ODP 1018	16	37	-123.6	n/a	1	1	terrestrial pollen in ocean sediments	C	tuned to LR04 stack	~500 cal yr (n = 0)	Lyle et al., 2012
Las Vegas Valley wetlands, NV	17	36.35	-115.25	750	2	3	wetlands sedimentology and dating, outcrop-based	D	^{14}C ages on mollusk shells, charcoal	260 cal yr (n = 15)	Springer et al., 2015
Panamint Lake, CA	18	36.33	-117.39	365	2	1	lake level ouctrop dating and core ostracod fauna	D	^{14}C ages on tufa, mollusk shells	260 cal yr (n = 3)	Jayko et al., 2008
Lake Estancia, NM	19	34.69	-105.95	1850	2	1	ostracode stratigraphy and shoreline dating	D	^{14}C ages on ostracodes, mollusk shells	270 cal yr (n = 11)	Allen and Anderson, 2000
ODP 893	20	34.3	-120	n/a	2	3	terrestrial pollen in ocean sediments	C	tuned to SPECMAP	~500 cal yr (n = 0)	Lyle et al., 2012
Lake Elsinore, CA	h	33.6733	-117.3542	379	2	3	lake sedimentology, stratigraphy	C	^{14}C ages on bulk sediment	400 cal yr (n = 11)	Kirby et al.,2013; 2018
Lake Cochise, AZ	22	32.15	-109.85	1262	2	1	lake level dating, outcrop-based	D	^{14}C ages on mollusk shells, charcoal	410 cal yr (n = 6)	Kowler et al., 2014
San Pedro Valley wetlands, AZ	23	31.75	-110.25	1100	2	1	wetlands sedimentology and dating, outcrop-based	D	^{14}C ages on mollusk shells	180 cal yr (n = 3)	Pigati et al., 2009

[a]Numbers 0-3 indicate differing relative wetness within each record for each time interval: 0 = no data, 1 = mostly drier, 2 = mostly wetter, 3 = variable wetness within the time interval, or not clearly conforming to the time interval boundaries

[b]Records are designated as continuous (core reconstructions) "C", or discontinuous (shorelines, spring discharge deposits) "D"

Table S2. ^{14}C ages for tufas, mollusk shells, and DIC in the Chewaucan lake system.

Sample ID	AMS Lab Number	Sample type	Latitude	Longitude	Elevation (m asl)	$\delta^{13}C$ (‰ VPDB)	Fraction Modern Carbon[a]	^{14}C age (^{14}C yr BP)	±1σ	^{14}C age±2σ (cal yr BP)	±2σ	Source
Water DIC												
CH13-24	AA104467	Abert Lake DIC	42.5755	-120.1896	1295	1.5	1.068	post-bomb	--	post-bomb	--	Hudson et al., 2017
Mollusk Shells												
CHL13-11a	AA102252	*Vorticifex effusa* shell	42.7567	-120.1867	1322	-1.6	0.229	11870	40	13670	100	this study
CHL13-11b	AA102253	*Vorticifex effusa* shell	42.7567	-120.1867	1322	-0.5	0.237	11570	40	13390	90	this study
CHL13-11c	AA102254	*Vorticifex effusa* shell	42.7567	-120.1867	1322	-1.4	0.235	11620	40	13460	100	this study
CHL13-12a	AA102255	*Vorticifex effusa* shell	42.7563	-120.1883	1346	-3.6	0.216	12310	60	14360	300	this study
CHL13-12b	AA102514	*Pyrgylopsis* sp. shell	42.7563	-120.1883	1346	0.4	0.224	12010	70	13900	180	this study
CHL13-13a	AA102256	*Vorticifex effusa* shell	42.7536	-120.1809	1309	-1.2	0.224	12030	80	13920	190	this study
CHL13-14-22	AA102257	*Vorticifex effusa* shell	42.7751	-120.1787	1326	2.8	0.251	11100	60	12930	180	this study
CHL13-14-40	AA102515	*Vorticifex effusa* shell	42.7751	-120.1787	1326	1.4	0.230	11820	60	13620	140	this study
CHL13-16b	AA102258	*Vorticifex effusa* shell	42.7594	-120.1616	1324	1.4	0.227	11920	60	13760	190	this study
CHL13-16c	AA102516	*Pyrgylopsis* sp. shell	42.7594	-120.1616	1324	0.1	0.211	12510	80	14690	420	this study
CHL13-19b	AA102259	*Vorticifex effusa* shell	42.7068	-120.1355	1303	-2.0	0.230	11800	60	13620	130	this study
CHL13-19d	AA102517	*Vorticifex effusa* shell	42.7068	-120.1355	1303	-1.7	0.227	11890	60	13750	190	this study
CHL14-32-5	AA106973	*Vorticifex effusa* shell	42.5350	-120.2293	1307	-5.1	0.228	11880	70	13720	230	this study
CHL15-56-1	AA106978	*Vorticifex effusa* shell	42.5981	-120.1856	1310	-3.5	0.224	11990	70	13880	170	this study
4F	AA13588	*Valvata humeralis* shell	42.5129	-120.3296	1325	--	--	11930	90	13790	230	Licciardi, 2001
6AV	AA13589	*Valvata humeralis* shell	42.5990	-120.1845	1310	--	--	11670	90	13520	200	Licciardi, 2001
7EG	AA13590	*Vorticifex effusa* shell	42.5351	-120.2280	1307	--	--	11560	120	13410	280	Licciardi, 2001
7FG	AA13591	*Vorticifex effusa* shell	42.5351	-120.2280	1310	--	--	12030	90	13880	170	Licciardi, 2001
ZX-1-1 shell	AA20142	shell, unknown type	--	--	1321	-1.8	0.2198	12170	110	14170	420	Friedel, 2001
ZX-1-2 shell	AA20143	shell, unknown type	--	--	1325	-0.8	0.2152	12340	80	14430	380	Friedel, 2001
Allison sample	--	shells, unknown type	--	--	1349	--	--	17500	300	21180	770	Allison, 1982
Tufas												
CHL13-2	AA104241	shorezone tufa	43.0477	-120.6815	1346	3.4	0.213	12440	40	14570	330	Hudson et al., 2017
CHL13-5	AA102250	shorezone tufa	43.0490	-120.6810	1346	3.3	0.217	12270	40	14220	190	Hudson et al., 2017
CHL13-18a	AA102518	shorezone tufa	42.7476	-120.1111	1322	3.8	0.153	15060	70	18290	220	this study
CHL13-19a	AA106475	shorezone tufa	42.7068	-120.1355	1304	4.0	0.112	17580	60	21240	250	this study
CHL13-19c	AA102519	shorezone tufa	42.7068	-120.1355	1309	1.3	0.306	9510	50	10840	240	this study
CHL13-21	AA102260	shorezone tufa	42.6394	-120.1843	1296	4.1	1.011	post-bomb	--	post-bomb	--	Hudson et al., 2017
CHL13-22	AA102261	shorezone tufa	42.6024	-120.1867	1299	3.7	0.164	14540	70	17720	210	Hudson et al., 2017
CHL14-27A	AA106476	shorezone tufa	42.7517	-120.1520	1314	3.5	0.305	9540	40	10900	190	this study
CHL14-27B-1	AA105807	shorezone tufa	42.7517	-120.1520	1314	4.1	0.302	9620	50	10980	190	this study
CHL14-27B-2	AA105808	shorezone tufa	42.7517	-120.1520	1314	4.3	0.345	8540	70	9520	150	this study
CHL14-28	AA105809	shorezone tufa	42.7556	-120.1555	1341	4.0	0.220	12180	60	14070	170	this study
CHL13-29-1	AA105810	shorezone tufa	42.7570	-120.1565	1340	3.6	0.217	12260	40	14180	340	Hudson et al., 2017
CHL14-29-2	AA105811	shorezone tufa	42.7570	-120.1565	1340	3.9	0.237	11570	60	13400	140	this study
CHL14-30	AA105816	shorezone tufa	42.5773	-120.1843	1303	1.9	0.151	15190	80	18460	210	Hudson et al., 2017

[a]Ratio of ^{14}C in the sample to ^{14}C in the standard, where FMC = 1 corresponds in age to AD 1950

Table S2 continued. ^{14}C ages for tufas, mollusk shells, and DIC in the Chewaucan lake system.

Sample ID	AMS Lab Number	Sample type	Latitude	Longitude	Elevation (m asl)	$\delta^{13}C$ (‰ VPDB)	Fraction Modern Carbon[a]	^{14}C age (^{14}C yr BP)	±1σ	^{14}C age±2σ (cal yr BP)	±2σ	Source
CHL14-28	AA105809	shorezone tufa	42.7556	-120.1555	1341	4.0	0.220	12180	60	14070	170	this study
CHL13-29-1	AA105810	shorezone tufa	42.7570	-120.1565	1340	3.6	0.217	12260	40	14180	340	Hudson et al., 2017
CHL14-29-2	AA105811	shorezone tufa	42.7570	-120.1565	1340	3.9	0.237	11570	60	13400	140	this study
CHL14-30	AA105816	shorezone tufa	42.5773	-120.1843	1303	1.9	0.151	15190	80	18460	210	Hudson et al., 2017
CHL14-31A	AA105817	shorezone tufa	42.5768	-120.1869	1297	3.9	0.888	960	30	860	70	Hudson et al., 2017
CHL14-31B-1	AA105818	shorezone tufa	42.5768	-120.1869	1297	4.7	0.641	3570	40	3870	110	this study
CHL14-31B-2	AA105819	shorezone tufa	42.5768	-120.1869	1297	4.4	0.736	2470	40	2570	150	this study
CHL14-32-1	AA105971	shorezone tufa	42.5350	-120.2293	1301	3.8	0.157	14850	90	18060	250	Hudson et al., 2017
CHL14-32-3b	AA106971	shorezone tufa	42.5350	-120.2293	1309	4.0	0.299	9710	70	11020	230	this study
CHL14-32-4	AA106972	shorezone tufa	42.5350	-120.2293	1305	3.4	0.213	12400	60	14500	360	this study
CHL15-49-1	AA106974	shorezone tufa	42.5232	-120.2367	1297	3.4	0.860	1230	30	1170	100	this study
CHL15-49-2	AA106975	shorezone tufa	42.5232	-120.2367	1297	4.1	1.029	post-bomb	--	post-bomb	--	Hudson et al., 2017
CHL15-51-1	AA106976	shorezone tufa	42.5232	-120.2354	1306	4.2	0.117	17110	80	20650	240	this study
CHL15-51-2	AA106977	shorezone tufa	42.5232	-120.2354	1306	2.7	0.155	14910	70	18130	200	this study
CHL15-56-2	AA106979	shorezone tufa	42.5981	-120.1856	1310	3.9	0.288	10070	50	11640	300	this study
CHL15-69-1	AA106981	shorezone tufa	42.6632	-120.1744	1297	4.0	0.312	9370	50	10580	150	this study
CHL15-61	AA106982	shorezone tufa	42.7818	-120.2233	1350	4.1	0.233	11670	60	13530	370	this study
CHL15-68-1	AA106983	shorezone tufa	42.6624	-120.1755	1295	3.9	0.885	1020	30	920	120	this study
CHL15-68-2	AA106980	shorezone tufa	42.6624	-120.1755	1295	3.7	0.988	80	30	140	120	Hudson et al., 2017
CHL15-70-1	AA106984	shorezone tufa	42.6266	-120.1862	1295	4.3	0.952	380	30	410	90	this study
CHL15-70-2	AA106985	shorezone tufa	42.6266	-120.1862	1295	3.6	1.169	post-bomb	--	post-bomb	--	Hudson et al., 2017
CHL15-80A top	AA106986	shorezone tufa	43.0231	-120.7036	1321	3.9	0.163	14570	50	17760	180	this study
CHL15-80b bottom	AA106987	shorezone tufa	43.0231	-120.7036	1321	3.6	0.137	16010	110	19300	300	this study
CHL15-81 base	AA106988	shorezone tufa	43.0231	-120.7043	1343	3.8	0.228	11850	30	13660	90	this study
CHL15-82 top	AA106989	shorezone tufa	43.0232	-120.7041	1338	4.1	0.227	11870	70	13700	220	this study
CHL15-85 base	AA106990	shorezone tufa	43.0502	-120.6587	1345	3.7	0.209	12510	50	14720	360	this study
CHL15-87 base	AA106992	shorezone tufa	43.0499	-120.6589	1327	3.7	0.223	12100	60	13950	170	this study
CHL15-86 top	AA106991	shorezone tufa	43.0501	-120.6588	1338	4.0	0.240	11520	40	13360	80	this study
CHL15-87 top	AA106993	shorezone tufa	43.0499	-120.6589	1327	3.9	0.250	11160	80	12990	180	this study
CH-10-1	AA25320	shorezone tufa	--	--	1310	3.4	0.2153	12340	90	14450	410	Friedel, 2001
CH-10-2	AA25321	shorezone tufa	--	--	1310	3.5	0.2111	12490	90	14670	430	Friedel, 2001
CH-10-3	AA25322	shorezone tufa	--	--	1321	3.8	0.2272	11910	100	13750	270	Friedel, 2001
CH-10-5	AA25323	shorezone tufa	--	--	1337	3.8	0.2232	12050	130	13910	350	Friedel, 2001
--	Beta82260	shorezone tufa	--	--	1329	--	--	11880	80	13720	230	Friedel, 2001
SL15AE05	D-AMS 013004	shorezone tufa	43.0502	-120.6587	1345	--	--	12490	50	14670	370	Egger et al., 2018
SL15AE06	D-AMS 013005	shorezone tufa	43.0501	-120.6585	1343	--	--	11570	40	13390	90	Egger et al., 2018
SL15JH05	D-AMS 013010	shorezone tufa	43.0499	-120.6592	1328	--	--	11870	40	13670	100	Egger et al., 2018
SL15AE08	D-AMS 013006	shorezone tufa	42.8436	-120.6148	1344	--	--	10980	40	12850	120	Egger et al., 2018
SLT3-1B	D-AMS 013015	shorezone tufa	43.0060	-120.7700	1316	--	--	12530	40	14790	320	Egger et al., 2018
SLT1-2C-1	D-AMS 013014	shorezone tufa	43.0251	-120.7033	1322	--	--	12480	40	14640	360	Egger et al., 2018
SL15JH04	D-AMS 16779	shorezone tufa	43.0499	-120.6589	1329	--	--	12310	40	14160	230	Egger et al., 2018
SL15BM03	D-AMS 16780	shorezone tufa	43.0500	-120.6585	1342	--	--	12040	40	13820	130	Egger et al., 2018
Gehr sample[d]	--	shorezone tufa	--	--	1315	--	--	9390	40	10620	110	Gehr, 1980

[a]Ratio of ^{14}C in the sample to ^{14}C in the standard, where FMC = 1 corresponds in age to AD 1950
